# Increased frequency of angiotensin converting enzyme D allele in Chinese Han patients with idiopathic pulmonary fibrosis: A systematic review and meta-analysis

**DOI:** 10.1097/MD.0000000000030942

**Published:** 2022-10-07

**Authors:** Xiaozheng Wu, Wen Li, Gao Huang, Zhenliang Luo, Yunzhi Chen

**Affiliations:** a Department of Preclinical medicine, Guizhou University of Traditional Chinese Medicine, Guiyang, China.

**Keywords:** angiotensin converting enzyme, gene polymorphism, idiopathic pulmonary fibrosis, IPF, meta-analysis

## Abstract

To explore the association between Angiotensin Converting Enzyme (ACE) insert(I)/defect(D) gene polymorphism and the susceptibility to idiopathic pulmonary fibrosis (IPF).

**Methods:**

Searching PubMed, EMbase, CENTRAL, MEDLINE, CBM, China National Knowledge Infrastructure, WanFang Database and VIP Chinese Science database through a computer and collect the literature from China and foreign countries published before January 22, 2022. Screen the literatures and extract data such as first author, year of publication, diagnostic criteria and gene frequency, and draw a funnel chart and perform Begg’s Test and Egger’s test to evaluate publication bias. The influence analysis was performed for heterogeneous results and at the same time, the trial sequential analysis (TSA) was also conducted to confirm the robustness of the meta-analysis results. Registration number: CRD42021259341.

**Results:**

There were a total of 4 literatures (4 studies conducted in the Chinese Han population), and a total of 292 IPF patients and 351 healthy controls were included in this study. The results showed that in the Chinese Han population, the ACE I/D gene polymorphism was associated with the susceptibility of IPF (D vs I: [odds ratio, OR] = 0.53, 95% confidence interval [95%CI] [0.42, 0.67], *P* < .00001; DD vs II: [OR] = 0.37, 95%CI [0.24, 0.57], *P* < .00001; DD vs II + ID:[OR] = 0.30, 95%CI [0.21, 0.43], *P* < .00001), and the angiotensin II (Ang Ⅱ) level of IPF patients was higher than that of the control group (mean difference [MD] = 14.29, 95%CI [11.20,17.37], *P* < .00001).The TSA also confirmed that D allele was closely related to the susceptibility of IPF.

**Conclusion:**

In the Chinese Han population, the D allele of the ACE I/D gene polymorphism is associated with the susceptibility of IPF.

## 1. Introduction

Idiopathic pulmonary fibrosis (IPF) was a disease with unknown causes, insidious onset, short course and high mortality.^[1,2]^ In recent years, the incidence of IPF has been increasing year by year, and it has developed from a rare disease in the 1960s to a common one now. At present, the cause of IPF is not yet clear, but environmental risk factors such as smoking^[3,4]^ and inhalation of wood and metal dust^[5]^ have been reported to be associated with the development of IPF. Studies have shown that increased oxidative stress caused by smoking affects IPF progression in both former and current smokers in a dose-dependent manner compared with nonsmokers.^[6]^ A recent meta-analysis confirmed an increased risk of IPF in smokers compared with never-smokers (odds ratio [OR] = 1.39, 95% confidence interval [95% CI] [1.01–1.91]).^[7]^ In addition to environmental risk factors such as smoking, genetic risk factors also play an important role in the occurrence and progression of IPF.^[8–10]^

Angiotensin converting enzyme (ACE) gene is polymorphic, it’s located in the long arm 2 region of chromosome 17 with 3 bands (17q23).^[11]^ The insertion (I)/deletion (D) polymorphism of ACE gene was identified by cDNA probe hybridization. It was marked by whether there was a DNA fragment composed of 287 bases in intron 16 and Alu repeats to determine whether ACE gene was I type or D type.^[12]^ Therefore, ACE gene has 2 types of alleles: 490 base insertion (insertion, allele I) and 190 base non insertion (deletion, allele D); and 3 genotypes: type II, DD and ID.^[12]^ In the Caucasian healthy population, the distribution frequencies of D allele and I allele were 54.0% and 46.0%, respectively, and the distribution frequencies of DD, ID, and II genotypes were 31%, 22.0%, and 47%, respectively.^[13]^ In contrast, the distribution frequencies of D allele and I allele were 69.0% and 31.0%, respectively, in Caucasian patients with pulmonary fibrosis, and the distribution frequencies of DD, ID, and II genotypes were 42%, 4.0%, and 54%, respectively.^[13]^ The distribution frequencies of D allele and I allele were 43.93% and 56.07%, respectively, in Chinese Han healthy population, and the distribution frequencies of DD, ID, and II genotypes were 18.69%, 50.47%, and 30.84%, respectively.^[14]^ In contrast, the distribution frequencies of D allele and I allele were 61.76% and 38.24%, respectively, in IPF patients of Chinese Han population, and the distribution frequencies of DD, ID, and II genotypes were 47.06%, 29.41%, and 23.53%, respectively.^[14]^ Therefore, the gene distribution frequencies of ACE (I/D) polymorphisms vary among different ethnic groups. However, in patients with pulmonary fibrosis in different ethnic groups, the distribution frequencies of D allele and DD genotype tended to increase.

ACE is a potential role in the pathogenesis of IPF. The I/D of ACE gene can increase/decrease the activity of ACE in plasma and tissue.^[15–18]^ ACE can not only act on pulmonary vascular endothelial cells, epithelial cells, fibroblasts, but also change pulmonary vascular tension and permeability, coagulation and fibrinolysis system, pulmonary fibrosis through renin angiotensin system, and affect the occurrence, prognosis and prognosis of IPF. Its catalytic production of Angiotensin II (Ang II) can promote cell regeneration and extracellular matrix formation, which is related to the formation of pulmonary interstitial fibrosis.^[19]^ Therefore, the relationship between this gene polymorphism and IPF susceptibility has been studied continuously in recent 10 years. At present, some relevant studies have been published, but no consistent conclusion has been reached, and there is no relevant meta-analysis to further study the correlation between them. Therefore, this study conducted a meta-analysis on the relationship between ACE I/D gene polymorphism and IPF susceptibility because meta-analysis can summarize and analyze the data with the same research purpose to increase the effectiveness of the test and to draw more objective and reliable conclusions.

## 2. Material and Methods

This study has been registered in PROSPERO (https://www.crd.york.ac.uk/prospero/), registration number: CRD42021259341.

### 2.1. Inclusion and exclusion criteria

#### 2.1.1. Inclusion criteria.

The case-control study on ACE I/D gene polymorphism and IPF susceptibility. The language is Chinese or English, and the description of detection methods and means is accurate; It conforms to the authoritative standards established by the Chinese Society of Respiratory Medicine^[2]^ or the American Thoracic Society/European Respiratory Society/Japanese Respiratory Society/Latin American Thoracic Society.^[1]^ The patients were not limited in gender, age, race and nationality, and other serious systemic diseases were excluded; The gene frequency data is complete and can be used to calculate the OR and 95% CI; The distribution of genotype frequency of all subjects conformed to Hardy-Weinberg equilibrium^[20]^; The score of Newcastle Ottawa scale (NOS)^[21]^ was more than 7.

#### 2.1.2. Exclusion criteria.

Conference reports, reviews and case reports; The study of allele frequency could not be obtained; Research based on pedigree; For the same study published for many times, only the one with the largest sample size and the most complete information was reserved.

### 2.2. Outcomes

The pre-specified primary outcomes was to investigate whether ACE I/D increased the risk of IPF in the entire population. The secondary outcomes was to determine whether there was a difference in the strength of the association between ACE I/D and IPF among different ethnic groups.

### 2.3. Retrieval strategy

PubMed, EMbase, CENTRAL, MEDLINE, CBM, China National Knowledge Infrastructure, WanFang Database and VIP Chinese Science database were searched to collect relevant literatures published before January 22, 2022 on ACE gene polymorphism and IPF susceptibility from China and abroad. Subject words and keywords were used to retrieve, combined with manual retrieval and literature tracing. Key words: “angiotensin converting enzyme” or “ACE,” “polymorphism,” “idiopathic pulmonary fibrosis” or “IPF.” The language was limited to Chinese and English. Table 1 shows the search strategies in PubMed.

**Table 1:** PubMed search strategy.

**Number**	**Search terms**
**#1**	Mesh descriptor: (Idiopathic pulmonary fibrosis) explode all trees
**#2**	((((((Pulmonary fibrosisor[Title/Abstract]) OR Pulmonary interstitial fibrosis [Title/Abstract]) OR Interstitial lung disease [Title/Abstract]) OR IPF [Title/Abstract])
**#3**	Or 1-2
**#4**	Mesh descriptor: (angiotensin converting enzyme) explode all trees
**#5**	((((((ACE[Title/Abstract]) OR angiotensin converting enzyme[Title/Abstract])
**#6**	Or 4-5
**#7**	Mesh descriptor: (polymorphism) explode all trees
**#8**	3 and 6 and 7

### 2.4. Literature screening and data extraction

Two relatively independent researchers (X-ZW and WL) conducted literature screening and data extraction. After excluding the studies that obviously did not meet the inclusion criteria, they further read the full text of the studies that might meet the inclusion criteria to determine whether they could really be included, and then cross checked them. In case of different opinions, they discussed and resolved or submitted to the third party (Y-ZC) for ruling. They try to contact the author of the original text by e-mail to further obtain the relevant data if the report is not clear or lack of information. The extracted data include: general clinical data, research subjects, year of publication, country of research subjects, race of research subjects, IPF diagnostic criteria, number of cases in case group and healthy control group, and frequency of each genotype.

### 2.5. Literature quality evaluation

Two independent researchers (X-ZW and WL) evaluated the quality of the selected literature according to NOS^[21]^ with a score ranged from 0 to 9. Literature with a score of more than 7 points is considered as high-quality.

### 2.6. Statistical methods

Revman5.3 and stata12.0 software were used for data processing; Hardy Weinberg genetic balance of healthy control group was analyzed by Pearson test; The heterogeneity between studies was assessed by *Q* test and *I*^2^. If *P* < .1 or *I*^2^ > 50%, the data were combined by random effect model, and OR or mean difference (MD) and 95% CI were calculated; If there is no heterogeneity between studies, fixed effect model will be used for data consolidation; The OR values were calculated according to allele genetic model (D vs I), dominant genetic model (DD + DI vs II), recessive genetic model (DD vs DI + II),additive genetic model (DD vs II) and heterozygous genetic model (DI vs II); MD value was calculated according to Ang Ⅱ data; The forest plot was drawn to show the research results and their characteristics; The publication bias was judged by funnel plot, and it was evaluated by Begg’s Test and Egger’s Test. Influence analysis was performed for the results with high heterogeneity. We used test sequence analysis (TSA) software (http://www.ctu.dk/tsa/) to perform TSA test to evaluate the robustness of the conclusion (Type I error) probability = 5%, statistical test power = 80%, relative risk reduction = 20%).

## 3. Results

### 3.1. Literature search results

**Figure 1.**
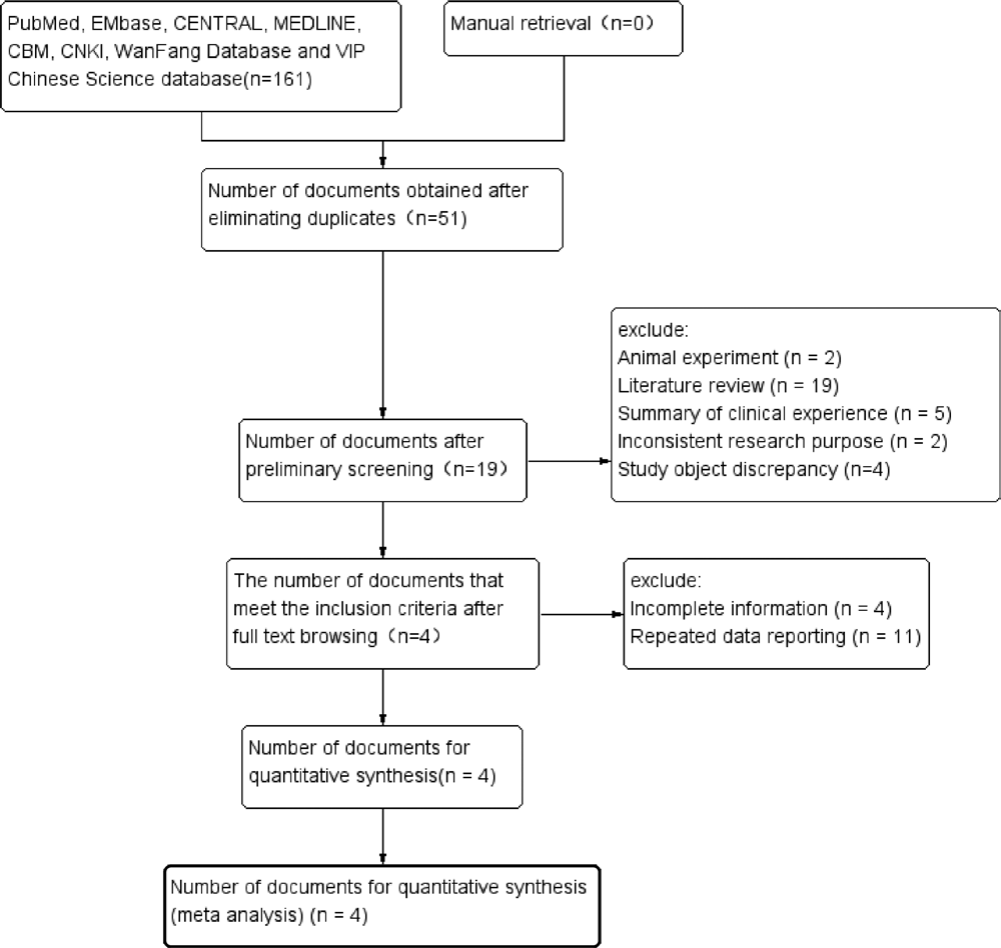
PRISMA literature screening flow chart (161 relevant literatures were initially found in the 5 databases and after tiered screening, 4 studies were finally included).

161 relevant literatures were initially found in the 8 databases. And after tiered screening, 4 studies were finally included.^[14,22–24]^ There were no English literatures (2 studies^[13,25]^ in Caucasians were excluded because they did not meet the inclusion criteria). A total of 292 IPF patients and 351 healthy controls were included. Figure 1 is the flow chart of literature screening developed by PRISMA statement,^[26]^ and Tables 2 and 3 are the basic characteristics of the included studies.

**Table 2:** Basic features of the included study (1).

Studies	Yr	Country	Nation	Diagnostic criteria	IPF group(n）	Control group(n）	Gender (male/female) (n）		Age (yrs)	
							IPF group	Control group	IPF group	Control group
Sun^[22]^	2010	China	HAN	Chinese Medical Association Guideline	64	54	36/28	32/22	58.4 ± 7.3	55.8 ± 4.3
You^[14]^	2013	China	HAN	ATS/ERS/JRS/ALAT Clinical Practice Guideline	102	107	56/46	58/49	61.4 ± 7.2	60.8 ± 4.3
Yu^[23]^	2010	China	HAN	Chinese Medical Association Guideline	42	90	29/13	57/33	52 ± 10	52 ± 9
Yuan^[24]^	2013	China	HAN	ATS/ERS/JRS/ALAT Clinical Practice Guideline	84	100	51/33	53/47	66 ± 10	50 ± 8

ALAT = Latin American Thoracic Society, ATS = American thoracic society, ERS = European respiratory society, IPF = idiopathic pulmonary fibrosis, JRS = Japanese respiratory society.

**Table 3:** Basic features of the included study (2).

Studies	Genotyping methods	IPF group						Control group						Hardy-Weinberg	
		II	ID	DD	I	D	DAF	II	ID	DD	I	D	DAF	χ^2^	*PHWE*
Sun^[22]^	PCR	13	19	32	45	83	64.84%	13	31	10	57	51	47.22%	0.193	*0.661*
You^[14]^	PCR	24	30	48	78	126	61.76%	33	54	20	120	94	43.93%	0.022	*0.883*
Yu^[23]^	PCR	13	17	12	43	41	48.81%	45	31	14	121	59	32.78%	0.356	*0.551*
Yuan^[24]^	PCR	21	23	40	65	103	61.31%	23	53	24	99	101	50.50%	0.110	*0.741*

DAF = D allele frequency, IPF = idiopathic pulmonary fibrosis, PCR = polymerase chain reaction, PHWE = *P* value of Hardy Weinberg equilibrium.

### 3.2. Quality evaluation

Four trials in this study were evaluated by NOS.^[21]^ The score of all trials results were high (≥7), indicating that the risk of bias was low. The results are shown in Table 4.

**Table 4:** Newcastle Ottawa scale (NOS).

**Studies**	**Select**				**Comparability** *	**Expose**			**Total score** †
	1	2	3	4	5	6	7	8	
	Ⅰ	ⅠⅠ	Ⅲ	Ⅳ	Ⅴ	Ⅵ	Ⅶ	Ⅷ	
Sun 2010^[22]^	☆☆	☆	☆	☆	☆		☆		7☆
You 2013^[14]^	☆☆	☆	☆	☆	☆		☆		7☆
Yu 2010^[23]^	☆☆	☆	☆	☆	☆		☆		7☆
Yuan 2013^[24]^	☆☆	☆	☆	☆	☆		☆		7☆

*Note*: 1-8: Case-control studies (CC);Ⅰ-Ⅷ:Cohort studies (CS).

1. Case definition;2: Case manifestations;3. Selection of control group;4. Definition of control group;5: Choose the most important/second most important factor;6. Determination of exposure;7. Methods for determining cases and control groups; 8: No response rate; I: representativeness of exposure; II: selection of non exposed persons; III: Determination of exposure; IV: proof of no interesting results at the beginning; V: comparability; VI: evaluation of results; VII: long enough follow-up time; VIII: adequacy of follow-up.

* Two stars with the highest comparability.

† Full score is 9☆.

### 3.3. Meta analysis

#### 3.3.1. D versus I.

**Figure 2.**
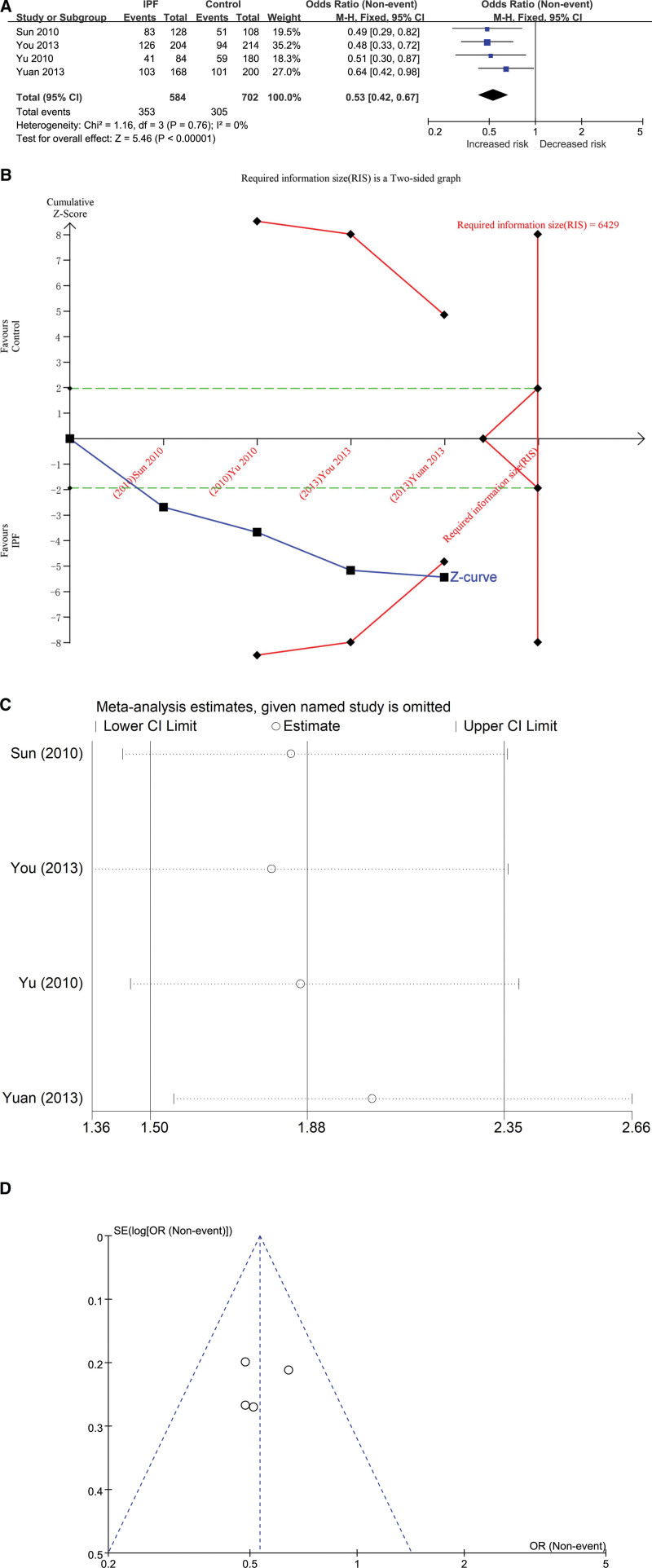
The association between ACE gene I/D polymorphism and IPF susceptibility was evaluated by allele genetic model (D vs I). (A) Forest plot of D versus I genetic model (The D allele was correlated with the risk of IPF in the Chinese Han population [OR] = 0.53, 95% CI [0.42, 0.67], *P* < .00001). (B) Trial sequential analysis (Adjusted Boundaries Print) of ACE I/D polymorphism and IPF risk using the D versus I genetic model. The calculated Required information size (RIS) was 6429, and the cumulative Z curve crossed the conventional boundary and the Trial sequential monitoring (TSA) boundary. The Blue line was cumulative Z curve; The Red line was the TSA boundary or RIS. The Green line was the Conventional boundary. (C) Influence analysis results of D versus I (The minimum of all the research results was not lower than the number 1). (D) Inverted funnel plot of D versus I (The inverted funnel plot showed symmetry, indicating that there was no obvious bias). 95% CI = 95% confidence interval, ACE = angiotensin converting enzyme, I/D = insert/defect, IPF = idiopathic pulmonary fibrosis, OR = odds ratio.

The association between ACE gene I/D polymorphism and IPF susceptibility was evaluated by allele genetic model. The total number of cases in IPF group and control group were 584 and 702 respectively after combining the studies. The heterogeneity test results showed that *P* = .76 and *I*^2^ = 0%, and the D allele was correlated with the risk of IPF in the Chinese Han population (OR = 0.53, 95% CI [0.42, 0.67], *P* < .00001) (Fig. 2A). In the TSA, the calculated required information size (RIS) was 6429. Although the combined sample size did not exceed the RIS, the cumulative Z curve crossed the conventional boundary and the TSA boundary, and the association was established in advance (Fig. 2B/Fig. S1 in supplemental content, http://links.lww.com/MD/H463), all of which indicate that no further research is needed, because such a significant association is unlikely change. Influence analysis results showed that the minimum of all the research results was not lower than the number 1, indicating that there was no significant difference in the results after removing any one of the studies (Fig. 2C/Table S1 in supplemental content, http://links.lww.com/MD/H488). The test results of the inverted funnel plot showed symmetry, indicating that there was no obvious bias (Fig. 2D). The results of Begg’s Test (*P* = .734) and Egger’s Test (*P* = .480) suggested that there was no obvious bias (Table S2, http://links.lww.com/MD/H489, Table S3, http://links.lww.com/MD/H490, Figs. S2, http://links.lww.com/MD/H464 and S3, http://links.lww.com/MD/H465 in supplemental content）.

#### 3.3.2. DD + ID versus II

**Figure 3.**
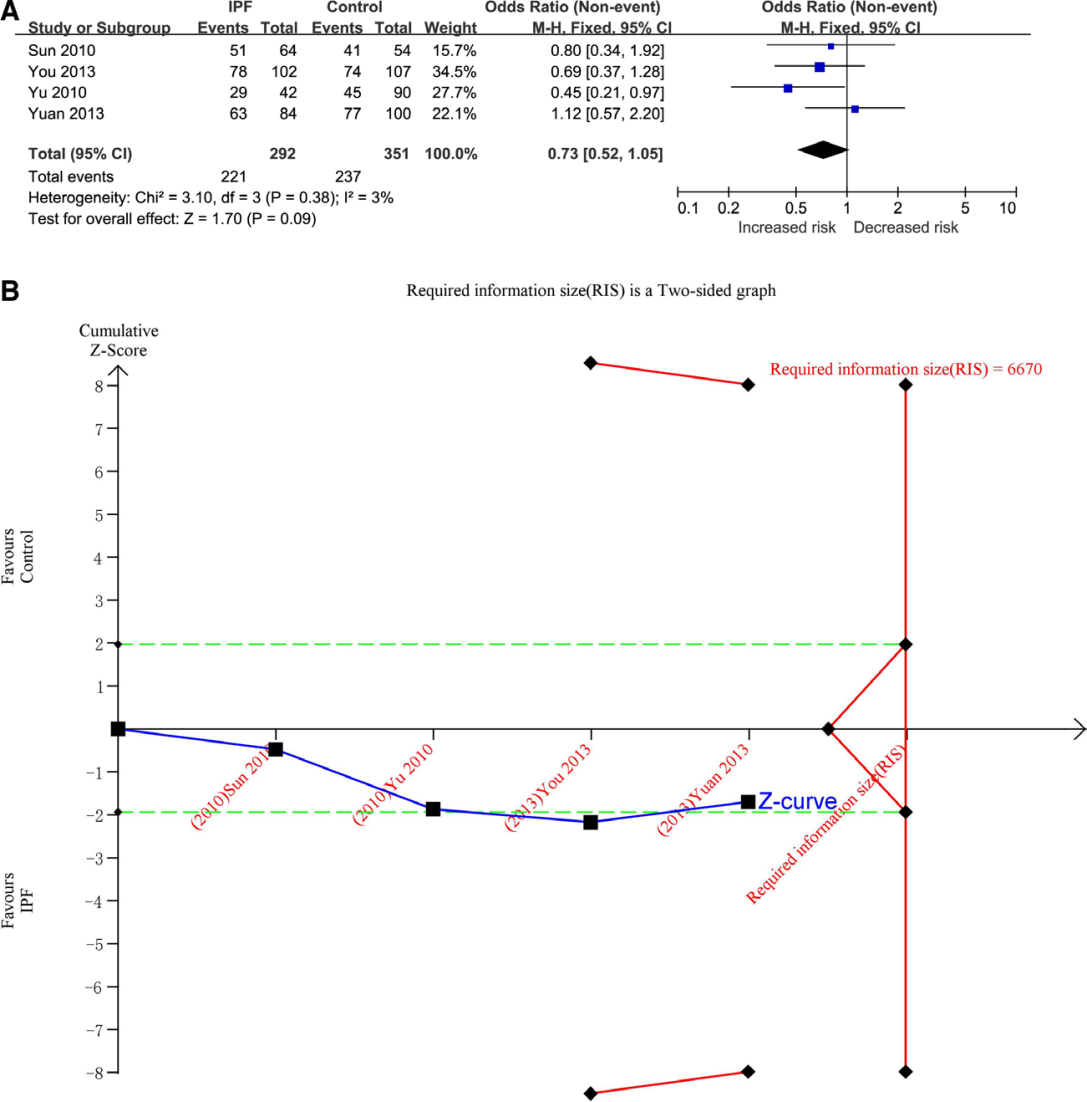
The association between ACE gene I/D polymorphism and IPF susceptibility was evaluated by dominant genetic model (DD + ID vs II). (A) Forest plot of DD + ID versus II genetic model. The DD + ID genotype was not correlated with the risk of IPF (OR = 0.73, 95% CI [0.52, 1.05], *P* = .09). (B) Trial sequential analysis (Adjusted Boundaries Print) of ACE I/D polymorphism and IPF risk using the DD + ID versus II genetic model. The calculated RIS was 6670 and the cumulative Z curve crossed the conventional boundary. The Blue line is cumulative Z curve; The Red line is the Trial sequential monitoring (TSA) boundary or Required information size (RIS); The Green line is the Conventional boundary. 95% CI = 95% confidence interval, ACE = angiotensin converting enzyme, I/D = insert/defect, IPF = idiopathic pulmonary fibrosis, OR = odds ratio.

The dominant genetic model was used to evaluate the correlation. There were 292 cases in the IPF group and 351 cases in the control group, respectively. The heterogeneity test showed that *P* = .38 and *I*^2^ = 3%, and the DD + ID genotype was not correlated with the risk of IPF (OR = 0.73, 95% CI [0.52, 1.05], *P* = .09) (Fig. 3A). In TSA, the calculated RIS was 6670. The combined sample size did not exceed RIS, and the cumulative Z curve did not cross the TSA boundary, but it crossed the conventional boundary (Fig. 3B/Fig. S4, http://links.lww.com/MD/H466 in supplemental content), indicating that a larger sample size was needed to confirm the correlation between DD + ID genotype and IPF risk. The influence analysis results showed that: the sensitivity of yuan (2013)^[24]^ of one study was higher, indicating that due to this study, the results may have differences, which proved that the effect of DD + ID versus II had certain sensitivity, and there was a certain dispute about stability and reliability (Table S4, http://links.lww.com/MD/H491, Fig. S5, http://links.lww.com/MD/H467 in supplemental content).The inverted funnel plot test results seem to be asymmetric, suggesting a certain degree of bias ((Fig. S6, http://links.lww.com/MD/H468 in supplemental content）, but the results of Begg’s Test (*P* = 1.000) and Egger’s Test (*P* = .804) suggested that there was no bias (Table S5, http://links.lww.com/MD/H492, Table S6, http://links.lww.com/MD/H493, Figs. S7, http://links.lww.com/MD/H469 and S8, http://links.lww.com/MD/H470 in supplemental content）.

#### 3.3.3. DD versus II + ID

**Figure 4.**
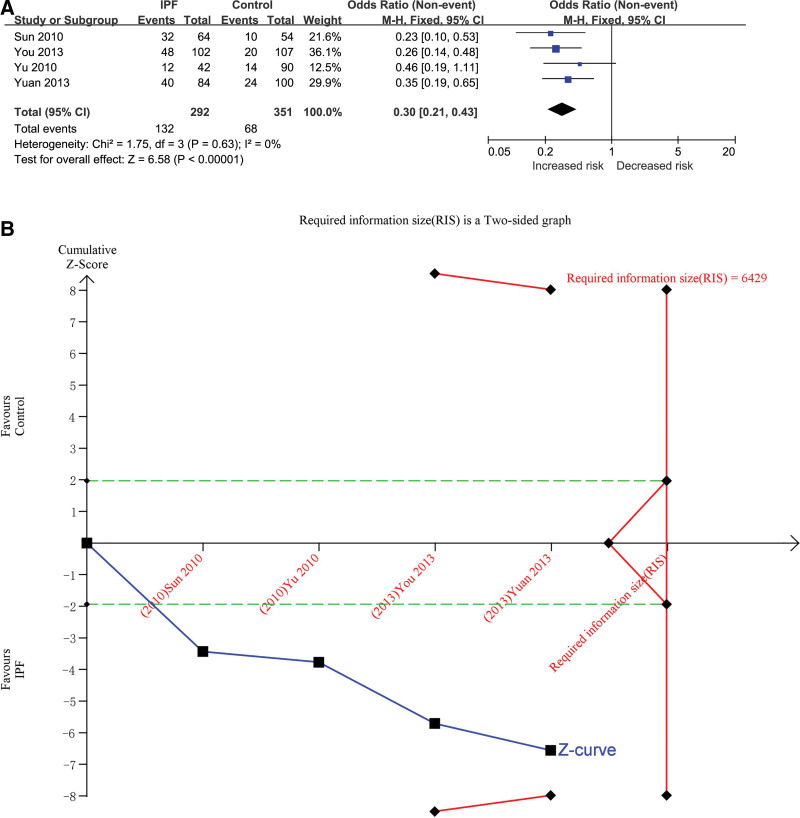
The association between ACE gene I/D polymorphism and IPF susceptibility was evaluated by recessive genetic model (DD vs II + ID). (A) Forest plot of DD versus II + ID genetic model. The DD genotype was correlated with the risk of IPF (OR = 0.30, 95% CI [0.21, 0.43], *P* < .00001). (B) Trial sequential analysis (Adjusted Boundaries Print) of ACE I/D polymorphism and IPF risk using the DD versus II + ID genetic model. The calculated RIS was 6429 and the cumulative Z curve crossed the conventional boundary. The Blue line is cumulative Z curve; The Red line is the Trial sequential monitoring (TSA) boundary or Required information size (RIS); The Green line is the Conventional boundary). 95% CI = 95% confidence interval, ACE = angiotensin converting enzyme, I/D = insert/defect, IPF = idiopathic pulmonary fibrosis, OR = odds ratio.

The recessive genetic model was used to evaluate the correlation. The total number of cases in IPF group and control group were 292 and 351, respectively, after combining the studies. The heterogeneity test results showed that *P* = .63 and *I*^2^ = 0%, and the DD genotype was correlated with the risk of IPF (OR = 0.30, 95% CI [0.21, 0.43], *P* < .00001) (Fig. 4A). In TSA, the calculated RIS was 6429. The combined sample size did not exceed RIS, and the cumulative Z curve did not cross the TSA boundary, but it crossed the conventional boundary (Fig. 4B/Fig. S9, http://links.lww.com/MD/H471 in supplemental content), indicating that a larger sample size is needed to confirm the correlation between DD genotype and IPF risk. Influence analysis results showed that the minimum of all the research results was not lower than the number 1, indicating that there was no significant difference in the results after removing any one of the studies (Table S7, http://links.lww.com/MD/H494, Fig. S10, http://links.lww.com/MD/H472 in supplemental content).The test results of the inverted funnel plot showed symmetry, indicating that there was no obvious bias (Fig. S11, http://links.lww.com/MD/H473 in supplemental content）.The results of Begg’s Test (*P* = .734) and Egger’s Test (*P* = .787) suggested that there was no obvious bias (Table S8, http://links.lww.com/MD/H495, Table S9, http://links.lww.com/MD/H496, Figs. S12, http://links.lww.com/MD/H474 and S13, http://links.lww.com/MD/H475 in supplemental content）.

### 3.3.4. DD versus II

**Figure 5.**
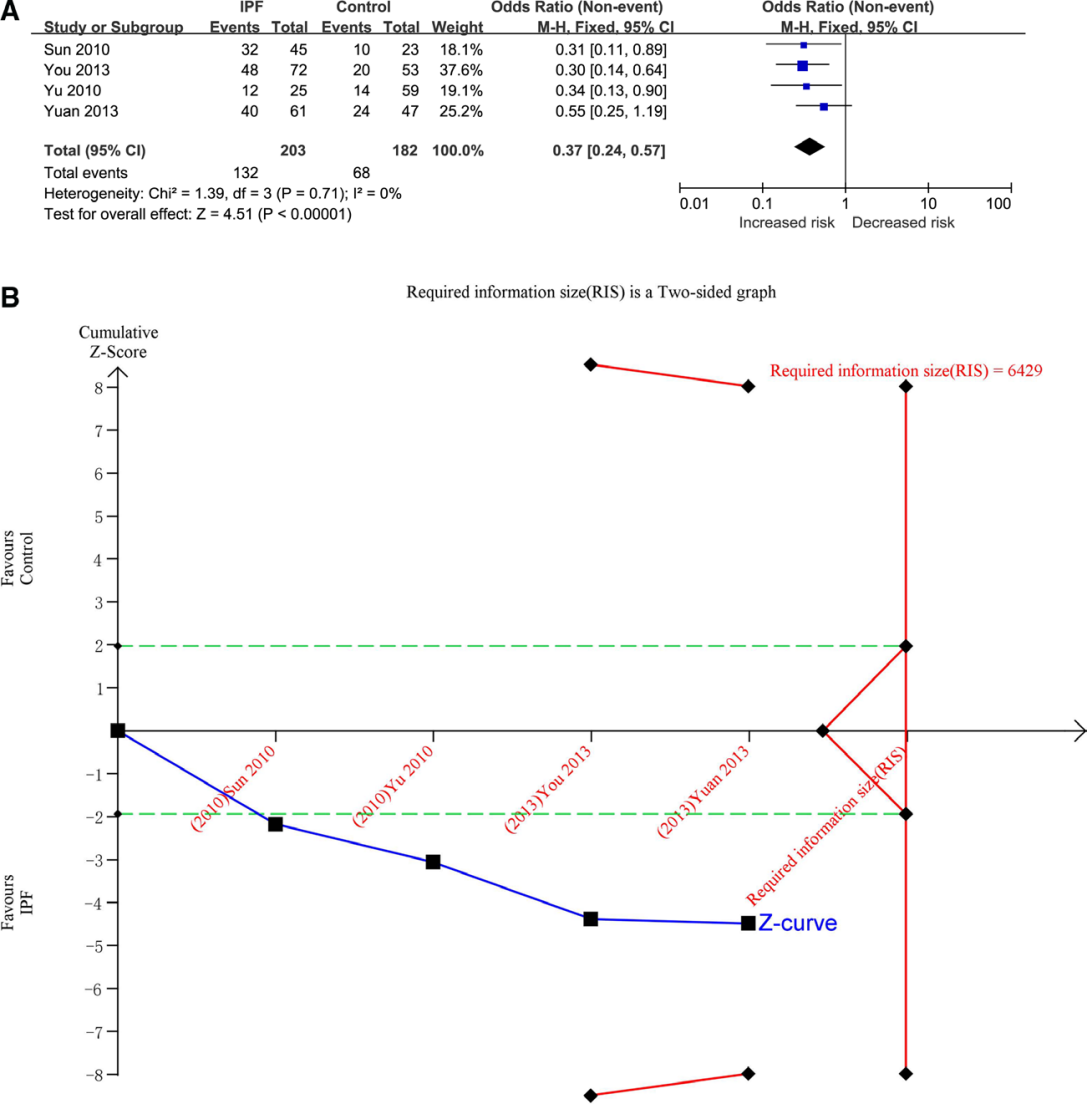
The association between ACE gene I/D polymorphism and IPF susceptibility was evaluated by additive genetic model (DD vs II). (A) Forest plot of DD versus II genetic model. The DD genotype was correlated with the risk of IPF (OR = 0.37, 95% CI [0.24, 0.57], *P* < .00001). (B) Trial sequential analysis (Adjusted Boundaries Print) of ACE I/D polymorphism and IPF risk using the DD versus II genetic model. The calculated RIS was 6429 and the cumulative Z curve crossed the conventional boundary. The Blue line is cumulative Z curve; The Red line is the Trial sequential monitoring (TSA) boundary or Required information size (RIS); The Green line is the Conventional boundary. 95% CI = 95% confidence interval, ACE = angiotensin converting enzyme, I/D = insert/defect, IPF = idiopathic pulmonary fibrosis, OR = odds ratio.

The additive genetic model was used to evaluate the correlation. The total number of cases in IPF group and control group were 203 and 182, respectively, after combining all studies. The heterogeneity test results showed that *P* = .71, *I*^2^ = 0%, and the DD genotype was correlated with the risk of IPF (OR = 0.37, 95% CI [0.24, 0.57], *P* < .00001) (Fig. 5A). In TSA, the calculated RIS was 6429. The combined sample size did not exceed RIS, and the cumulative Z curve did not cross the TSA boundary, but it crossed the conventional boundary (Fig. 5B/Fig. S14, http://links.lww.com/MD/H477 in supplemental content）, indicating that a larger sample size is needed to confirm the correlation between DD genotype and IPF risk. Influence analysis results showed that the minimum of all the research results was not lower than the number 1, indicating that there was no significant difference in the results after removing any one of the studies (Table S10, http://links.lww.com/MD/H497, Fig. S15, http://links.lww.com/MD/H479 in supplemental content).The test results of the inverted funnel plot showed symmetry, indicating that there was no obvious bias(Fig. S16, http://links.lww.com/MD/H480 in supplemental content).The results of Begg’s Test (*P* = 1.000) and Egger’s Test (*P* = .715) suggested that there was no obvious bias (Table S11, http://links.lww.com/MD/H498, Table S12, http://links.lww.com/MD/H499, Figs. S17, http://links.lww.com/MD/H481 and S18, http://links.lww.com/MD/H482 in supplemental content).

#### 3.3.5. ID versus II

**Figure 6.**
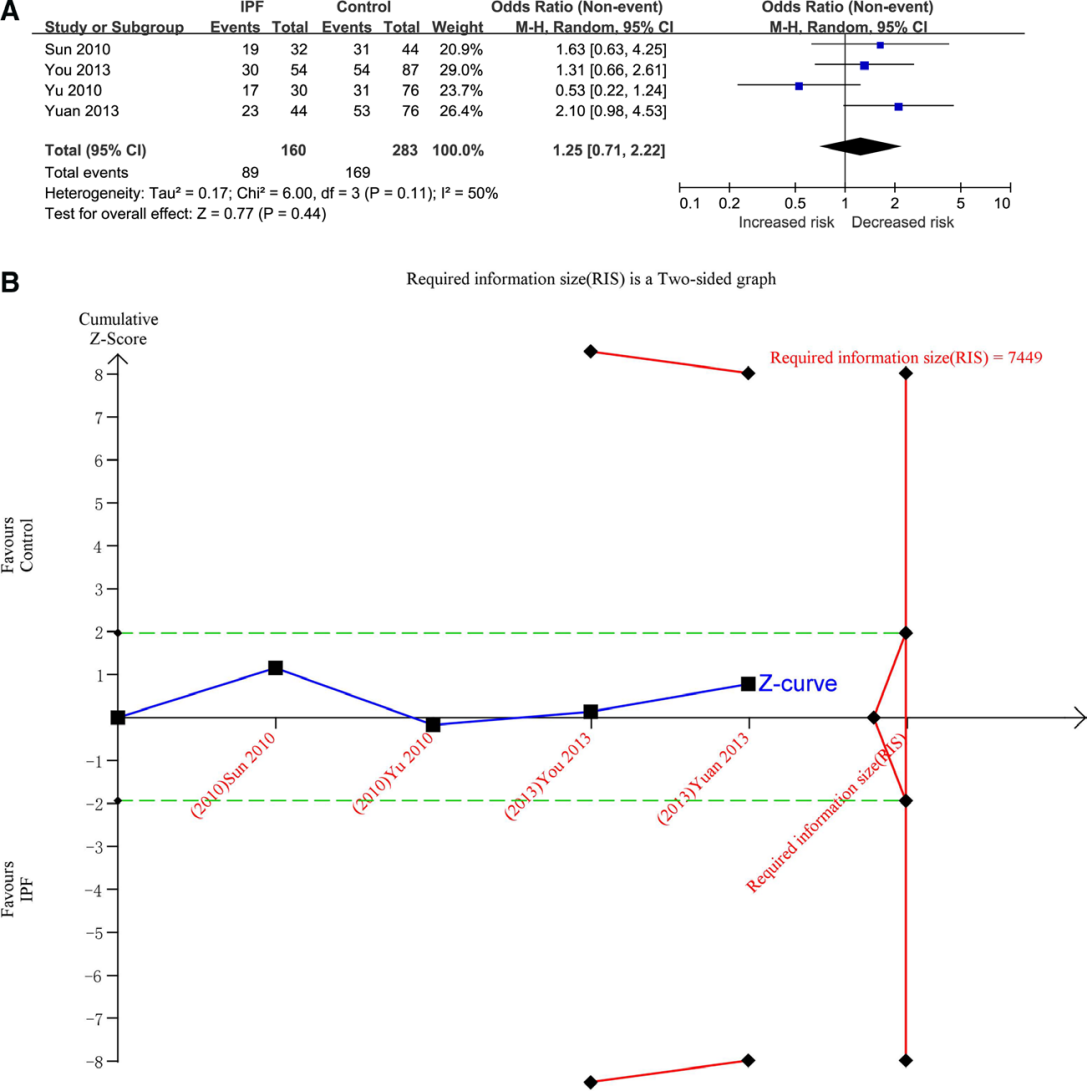
The association between ACE gene I/D polymorphism and IPF susceptibility was evaluated by heterozygous genetic model (ID vs II). (A) Forest plot of ID versus II genetic model. There was no correlation between ID genotype and the risk of IPF (OR = 1.25, 95% CI [0.71, 2.22], *P* = .44). (B) Trial sequential analysis (Adjusted Boundaries Print) of ACE I/D polymorphism and IPF risk using the ID versus II genetic model. The calculated RIS is 7449. The combined sample size did not exceed the RIS, and the cumulative Z curve did not cross the conventional boundary and the TSA boundary. The Blue line is cumulative Z curve; The Red line is the Trial sequential monitoring (TSA) boundary or Required information size (RIS); The Green line is the Conventional boundary. 95% CI = 95% confidence interval, ACE = angiotensin converting enzyme, I/D = insert/defect, IPF = idiopathic pulmonary fibrosis, OR = odds ratio.

The heterozygous genetic model was used to evaluate the correlation. The total number of cases in IPF group and control group were 160 and 283, respectively, after combining all studies. The heterogeneity test results showed that *P* = .11 and *I*^2^ = 50%, and there was no correlation between ID genotype and the risk of IPF (OR = 1.25, 95% CI [0.71, 2.22], *P* = .44) (Fig. 6A). In TSA, the calculated RIS is 7449. The combined sample size did not exceed the RIS, and the cumulative Z curve did not cross the conventional boundary and the TSA boundary (Fig. 6B/Fig. S19, http://links.lww.com/MD/H483 in supplemental content), indicating that the ID genotype has no correlation with the risk of IPF, and more trials are still needed to prove the correlation between them.

Due to the statistical heterogeneity of ID versus II heterozygous genetic model analysis results (*P* = .11, *I*^2^ = 50%), 3 methods were used for influence analysis: Change the statistical analysis model of combined effect size, the result showed no statistical change (*P* = .25); Remove the research method with the largest weight, the results showed that the effect size of the ID versus II heterozygous genetic model (*P* = .11, *I*^2^ = 50% or *P* = .11, *I*^2^ = 50%) did not change significantly before and after, and there was still no statistical significance (*P* = .41 or *P* = .44); Exclude the included studies one by one and observe the effect size and *P* changes. The results showed that the ID versus II heterozygous genetic model had *P* = .04, and the effect size changed significantly (*P* = .67, *I*^2^ = 0%) when the literature Yu (2010)^[23]^ was excluded, which proves that Yu (2010)^[23]^ is the main source of heterogeneity. Therefore, combining the results of the 3 analysis methods proves that the effect size of the ID versus II heterozygous genetic model has a certain degree of sensitivity, and its stability and reliability are somewhat controversial. The results of influence analysis in stata12.0 (Table S13, http://links.lww.com/MD/H500, Fig. S20, http://links.lww.com/MD/H484 in supplemental content) shows: Yu (2010)^[23]^ has high sensitivity, which indicates that there may be differences in the results due to this study, which further proves that the effect quantity of ID versus II heterozygous genetic model has a certain sensitivity, and its stability and reliability are controversial.

The inverted funnel plot analysis of the research results of ID versus II heterozygous genetic model showed that the inverted funnel plot was not symmetrical, indicating that there was a certain publication bias (Fig. S21, http://links.lww.com/MD/H485 in supplemental content). However, Begg’s Test (*P* = 1.000) and Egger’s test (*P* = .436) were later used to detect the results of the ID versus II heterozygous genetic model respectively, and the results showed that there was no obvious bias (Table S14, http://links.lww.com/MD/H501, Table S15, http://links.lww.com/MD/H502, Figs. S22, http://links.lww.com/MD/H486 and S23, http://links.lww.com/MD/H487 in supplemental content）.

#### 3.3.6. AngⅡ(ng/L)

**Figure 7.**
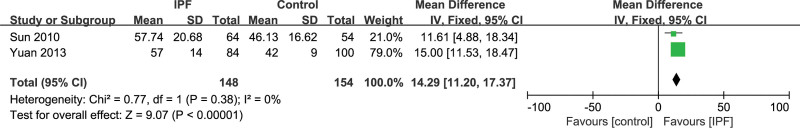
Forest plot of Ang Ⅱ changes. The level of Ang Ⅱ in IPF group was higher than that in control group (MD = 14.29, 95% CI [11.20, 17.37], *P < .00001*). 95% CI = 95% confidence interval, Ang II = angiotensin II, IPF = idiopathic pulmonary fibrosis, MD = mean difference.

Two studies^[22,24]^ reported the changes of Ang Ⅱ. The heterogeneity test results showed that *P* = .38 and *I*^2^ = 0%, and the level of Ang Ⅱ in IPF group was higher than that in control group (MD = 14.29, 95% CI [11.20, 17.37], *P* < .00001) (Fig. 7).

## 4. Discussion

The pathogenesis of IPF has not been fully elucidated. Environmental factors such as smoking have been found in numerous studies to increase the risk of IPF.^[27–30]^ In recent years, it has been reported that genetic genes may have a certain impact on the pathogenesis. ACE, also known as kinanese Ⅱ, is a zinc containing hydroxyl exohydrolase. Its main physiological function was to cut low activity angiotensin I into high activity 8 peptide Ang II,^[31–33]^ and Ang II was a powerful vasoconstrictor. The results showed that the changes of ACE gene expression in plasma were closely related to lung diseases, and can be used as an important indicator for diagnosis of lung diseases.^[34]^An important gene polymorphism of ACE gene is the I or D of a 287 bp DNA fragment in intron 16. Study had found that the D allele was associated with the increase of serum ACE level and activity.^[34]^Therefore, in recent 10 years, people have been studying the susceptibility between them. At present, some related studies have been published, but no consistent conclusion has been reached. Based on the meta-analysis, the data with the same research purpose can be summarized and analyzed, which increases the effectiveness of the test to draw more objective and reliable conclusions. Therefore, this study conducted a meta-analysis on the susceptibility between ACE I/ D gene polymorphism and IPF susceptibility.

This study included 4 case-control studies conducted in the Chinese Han population, including 292 patients with IPF and 351 healthy controls. The genotype and allele distribution frequency of all subjects in the control group were in accordance with Hardy Weinberg equilibrium test, which showed that the selection of the control group was representative of the population, and the samples were in a random distribution equilibrium and widely distributed population. The results showed that ACE I/D gene polymorphism was correlated with the risk of IPF in the genetic models of D versus I, DD versus II and DD versus II + ID in Chinese Han population, and the effect values were (OR = 0.53, 95% CI [0.42, 0.67], *P* < .00001), (OR = 0.37, 95% CI [0.24, 0.57], *P* < .00001) and (OR = 0.30, 95% CI [0.21, 0.43], *P* < .00001), moreover, the Ang Ⅱ level of IPF patients was higher than that of control group (MD = 14.29, 95% CI [11.20,17.37], *P* < .00001). Influence analysis and published bias analysis showed that the results were relatively stable and reliable. The results of the TSA also confirmed the risk of the D allele and IPF. It is suggested that the population with DD genotype and D allele is prone to pulmonary fibrosis. DD genotype and D allele are the risk factors of pulmonary fibrosis, and Ang Ⅱ level is high, which is consistent with the previous research results in Asian people.^[35]^ The results suggest that ACE I/ D gene polymorphism is one of the risk factors for IPF in Chinese Han population. The increased level of D allele can activate ACE and catalyze the production of Ang Ⅱ which leads to IPF. However, there are some differences in the results among Caucasians：McGrath^[25]^ found in a study on 118 cases of British and 56 cases of Czech Caucasian pulmonary nodules that ACE I/D gene frequency distribution was not related to the severity and progressive development of pulmonary fibrosis in Caucasians. In addition, Morrison^[13]^ found that the incidence of D allele and I allele in 24 white American patients with pulmonary fibrosis was 69.0% and 31.0%, respectively. Among the 48 observed alleles, D allele was statistically significant compared with the control group; DD genotype accounted for 42.0%, ID genotype accounted for 4.0%, II genotype accounted for 54.0%. There was no significant difference in the distribution of 3 genotypes between these patients and the control group. Compared with Morrison’s study, the distribution of D and I alleles was similar, but the distribution of DD genotype was different. The reason of the difference may be related to race difference, heredity and environment.

Heterogeneity is the main factor affecting the reliability of meta-analysis results. In this study, we found that there was a certain degree of heterogeneity and sensitivity among different studies in the ID versus II heterozygous genetic model when we combine the total effect values. The factors leading to heterogeneity may be the different environments of the subjects involved in different studies. In addition, there was a certain sensitivity in DD + ID versus II dominant genetic model, which also affects the credibility of this meta-analysis to a certain extent. And the comprehensiveness of data collection was affected since this study was only based on databases from China and abroad, and the retrieval languages were only set as Chinese and English. At the same time, because there were few studies abroad and few English literatures meeting the inclusion criteria of this study, only relevant data of Chinese Han population were collected, which affects the extrapolation of this meta-analysis. And the original studies included did not explore the content of gene-gene and gene-environment, thus relevant data couldn’t be collected, which makes it impossible to further analyze the interaction between the environment and gene. These all caused the limitations of this meta-analysis.In addition, the small sample size was also an important factor affecting the reliability of the results. TSA showed that the sample size of all genotypes did not reach RIS, which would inevitably lead to deviations and some false negative results. Therefore, a larger sample size will be still needed to confirm it.

## 5. Conclusions

Despite the shortcomings, this meta-analysis still overcame the limitations of small sample size, ethnic groups and regions in a single study, and reflected the relationship between them as much as possible. Although the meta-analysis results of this study showed that ACE I/D may be related to IPF susceptibility in Chinese Han population, and the D allele of the ACE I/D gene polymorphism is associated with the susceptibility of IPF, more and higher quality large sample case-control studies will be needed to verify this to provide more effective basis for the prevention and treatment of IPF.

## Authors contributions

This study is initiated by Xiaozheng Wu. Xiaozheng Wu will develop the search strategies, conduct data collection, and analyze independently. Zhenliang Luo, Gao Huang and Yunzhi Chen will revise it. All authors have approved the final manuscript.

**Conceptualization:** Xiaozheng Wu.

**Data curation:** Xiaozheng Wu.

**Formal analysis:** Xiaozheng Wu.

**Funding acquisition:** Xiaozheng Wu.

**Investigation:** Xiaozheng Wu.

**Methodology:** Xiaozheng Wu, Wen Li.

**Project administration:** Xiaozheng Wu.

**Resources:** Xiaozheng Wu.

**Software:** Xiaozheng Wu.

**Supervision:** Yunzhi Chen.

**Validation:** Xiaozheng Wu.

**Visualization:** Xiaozheng Wu.

**Writing – original draft:** Xiaozheng Wu.

**Writing – review & editing:** Xiaozheng Wu, Wen Li, Gao Huang, Zhenliang Luo.

## Supplementary Material


