# Supplementary material for: Increased frequency of angiotensin converting enzyme D allele in Chinese Han patients with idiopathic pulmonary fibrosis: A systematic review and meta-analysis
Source: Medicine (Baltimore). 2022 Oct 7;101(40):e30942. doi: 10.1097/MD.0000000000030942 (PMC9542842; doi:10.1097/MD.0000000000030942)

Figure S1 Trial sequential analysis of ACE I/D polymorphism and IPF risk using the allelic model (D vs.I)(Adjusted Boundaries Sketch).The Blue line was cumulative Z curve;The Red line was the Trial sequential monitoring(TSA) boundary or Required information size(RIS) ; The Green line was the Conventional boundary.

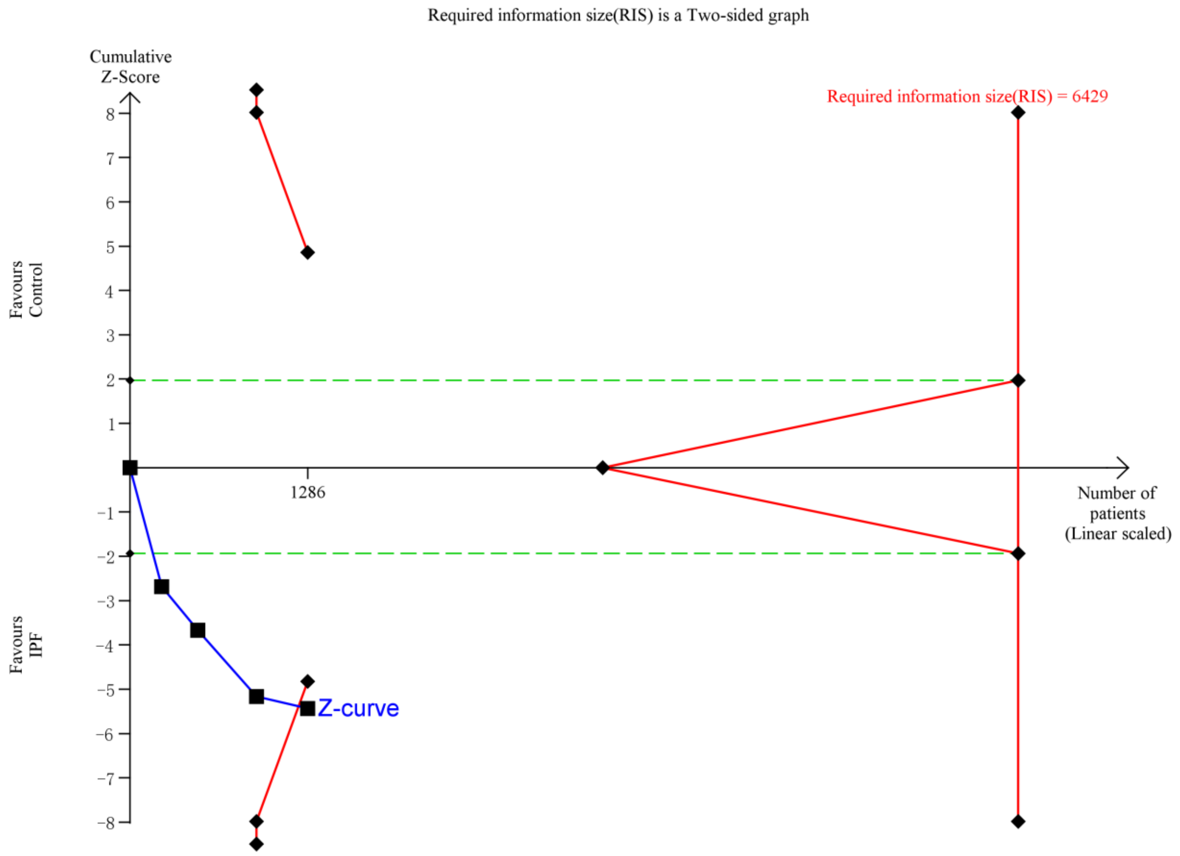

Supplement: Supplementary file 1 [file medi-101-e30942-s001.pdf]
