# Supplementary figures and images for: Increased frequency of angiotensin converting enzyme D allele in Chinese Han patients with idiopathic pulmonary fibrosis: A systematic review and meta-analysis
Source: Medicine (Baltimore). 2022 Oct 7;101(40):e30942. doi: 10.1097/MD.0000000000030942 (PMC9542842; doi:10.1097/MD.0000000000030942)

Figure S2 D vs.I funnel chart generated by Begg's Test

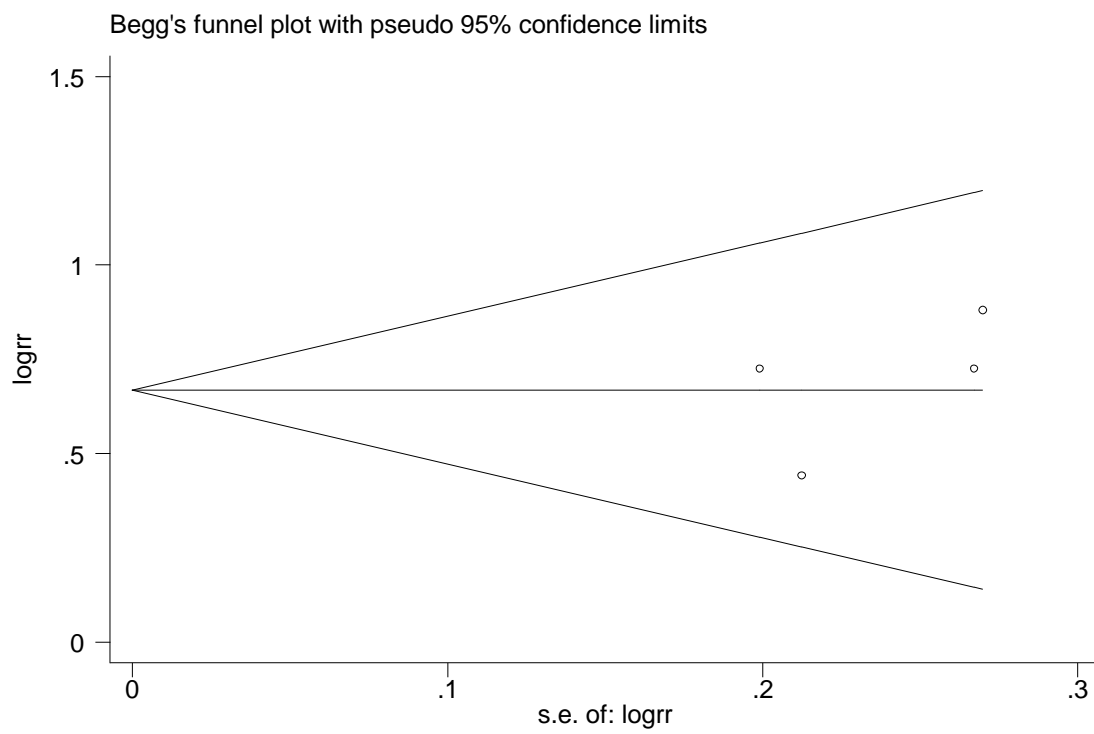

Supplement: Supplementary file 2 [file medi-101-e30942-s002.pdf]

Figure S3 D vs.I funnel chart of bias generation detected by Egger's test

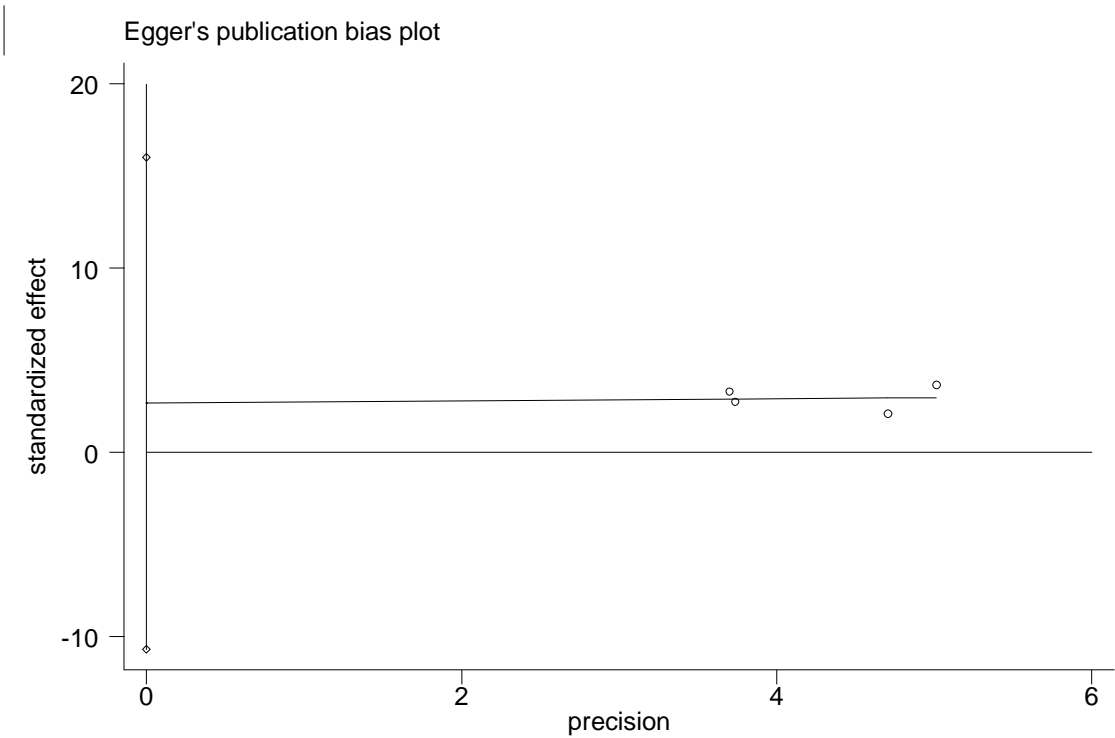

Supplement: Supplementary file 3 [file medi-101-e30942-s003.pdf]

Figure S5 Influence analysis results of DD+ID vs.II

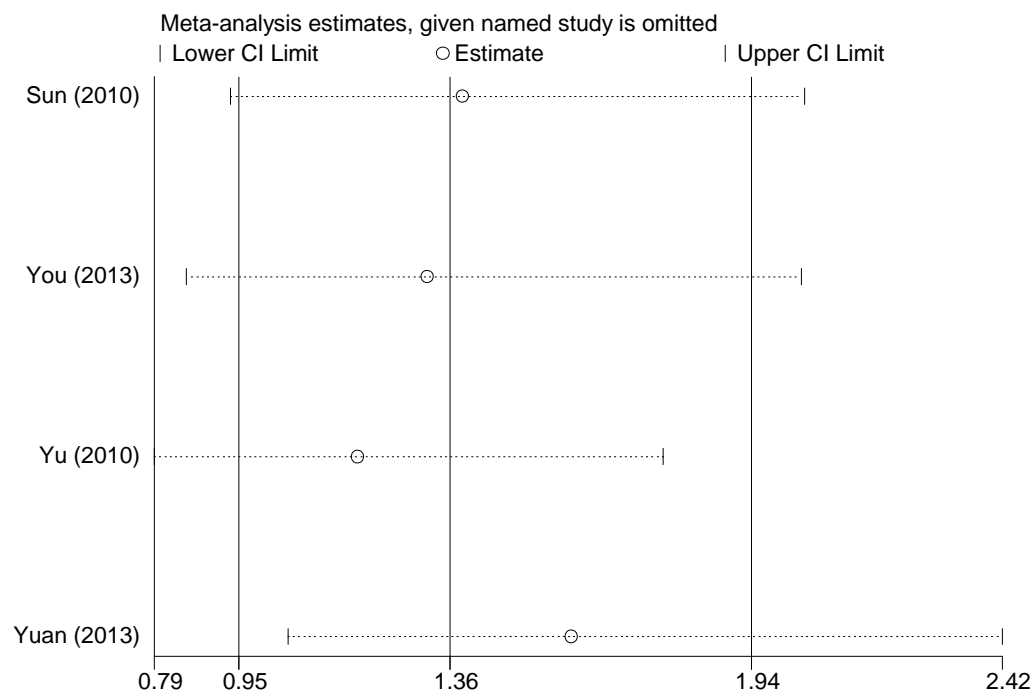

Supplement: Supplementary file 5 [file medi-101-e30942-s005.pdf]

Figure S6 Inverted funnel chart of DD+ID vs.II

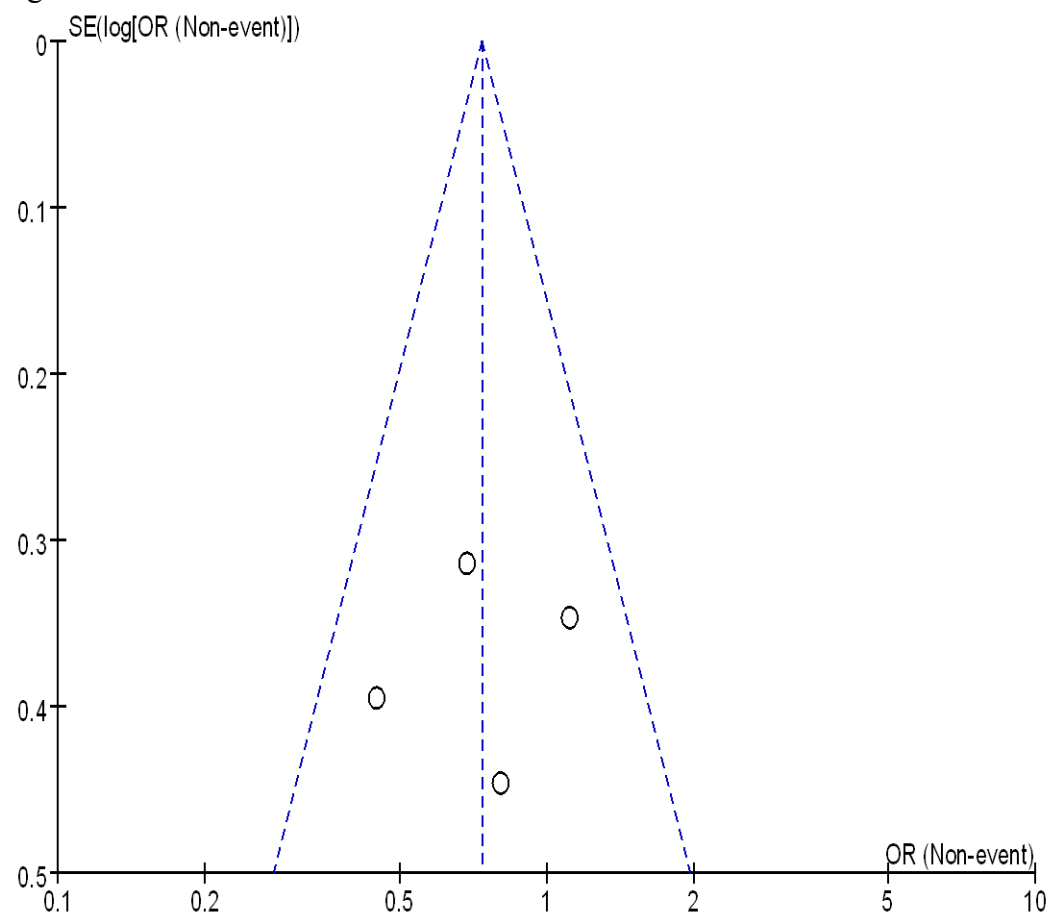

Supplement: Supplementary file 6 [file medi-101-e30942-s006.pdf]

Figure S7 DD+ID vs.II funnel chart generated by Begg's Test

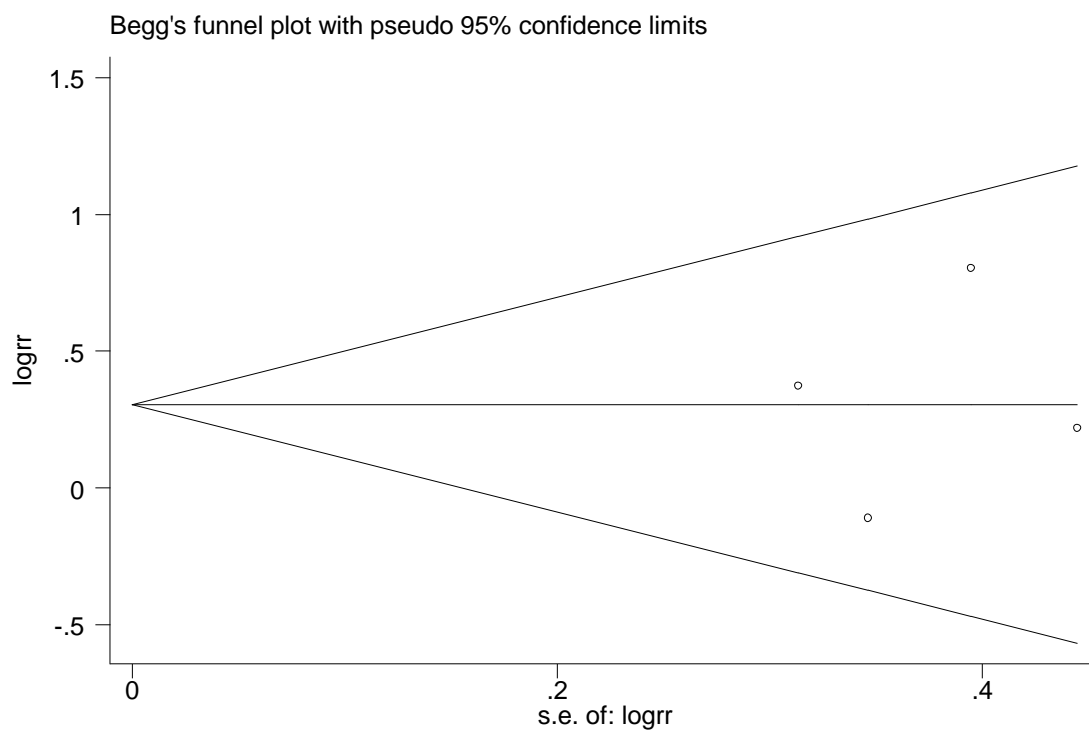

Supplement: Supplementary file 7 [file medi-101-e30942-s007.pdf]

Figure S8 DD+ID vs.II funnel chart of bias generation detected by Egger's test

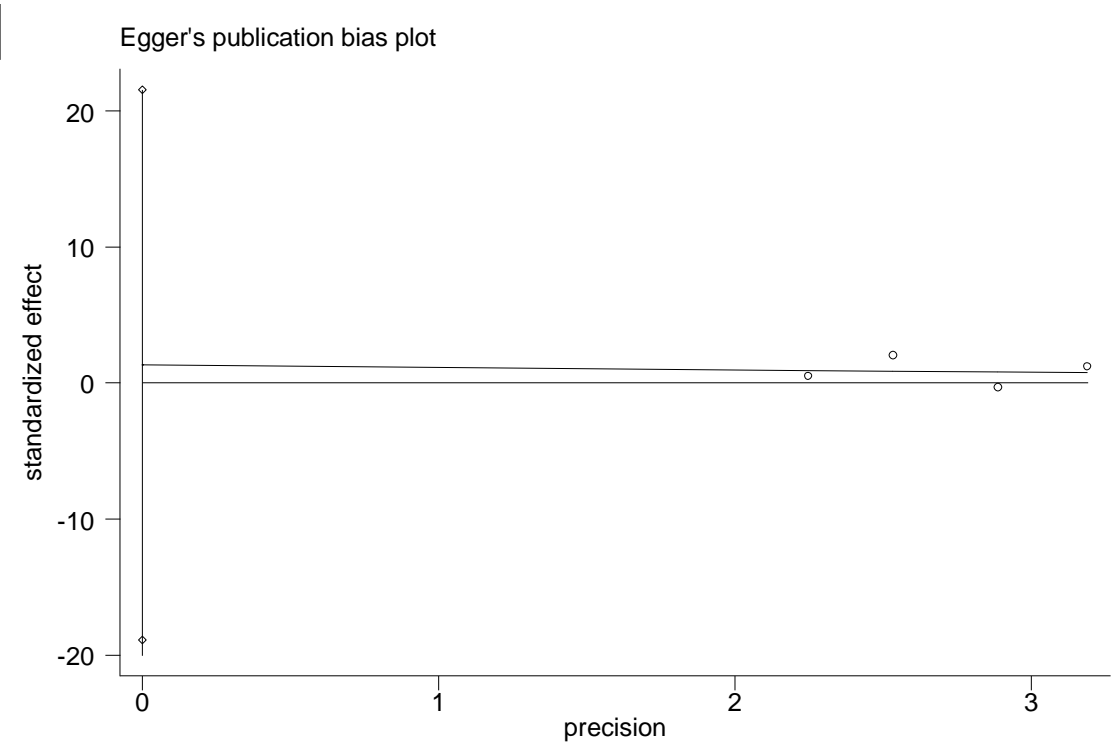

Supplement: Supplementary file 8 [file medi-101-e30942-s008.pdf]

Figure S10 Influence analysis results of DD vs. II+ID

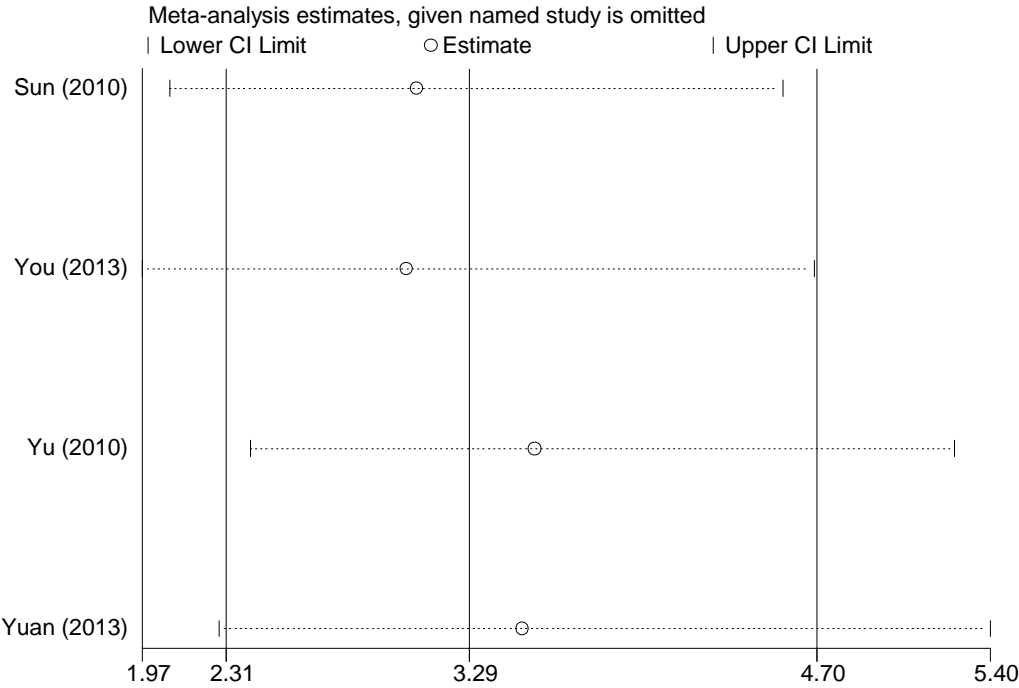

Supplement: Supplementary file 10 [file medi-101-e30942-s010.pdf]

Figure S11 Inverted funnel chart of DD vs. II+ID

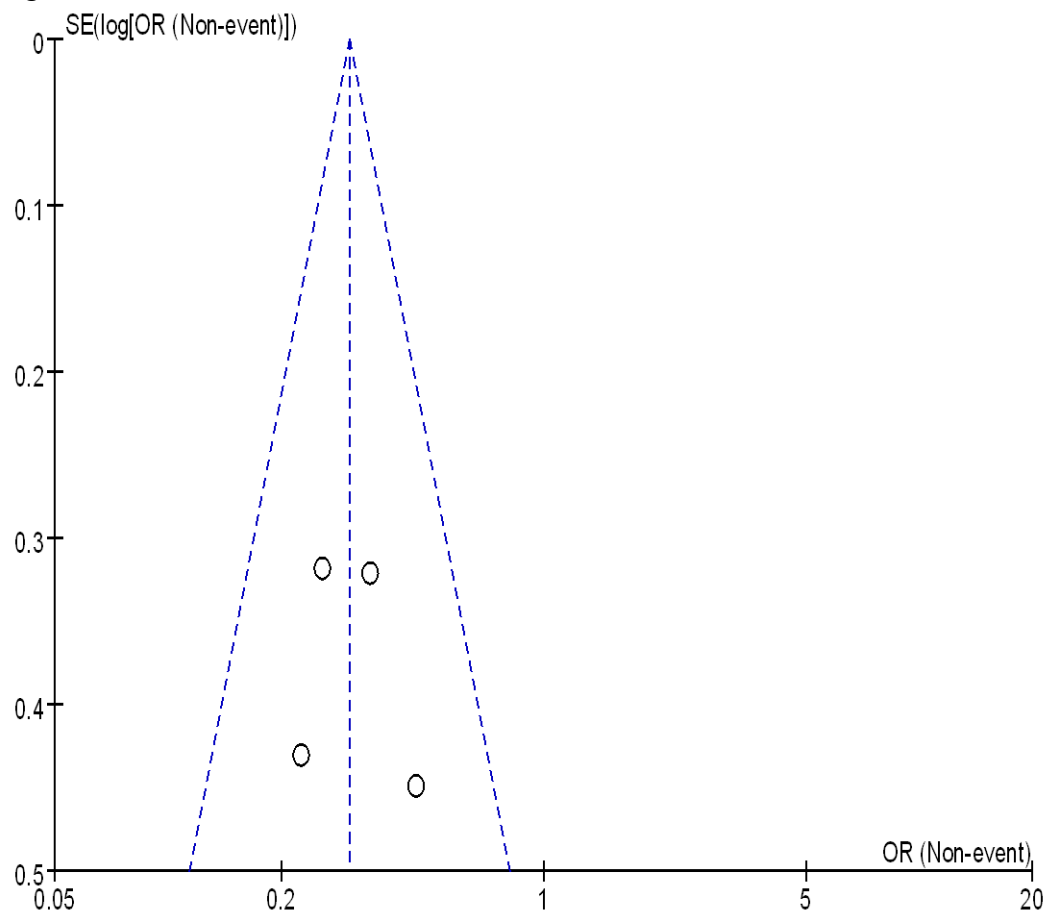

Supplement: Supplementary file 11 [file medi-101-e30942-s011.pdf]

Figure S12 DD vs. II+ID funnel chart generated by Begg's Test

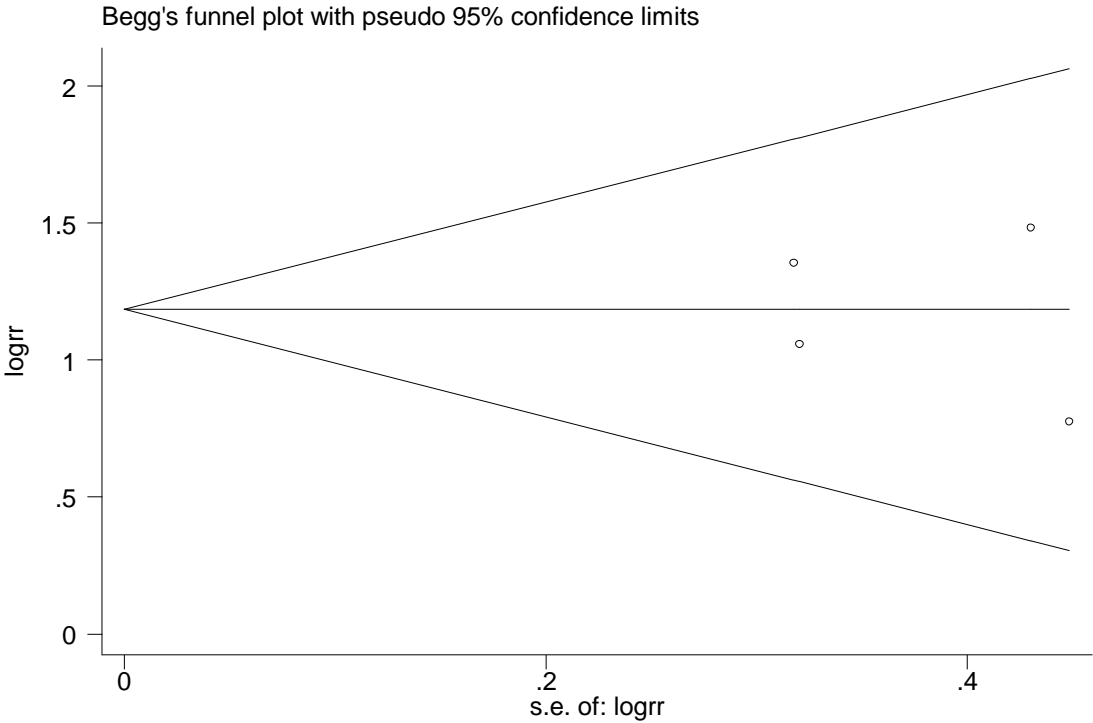

Supplement: Supplementary file 12 [file medi-101-e30942-s012.pdf]

Figure S13 DD vs. II+ID funnel chart of bias generation detected by Egger's test

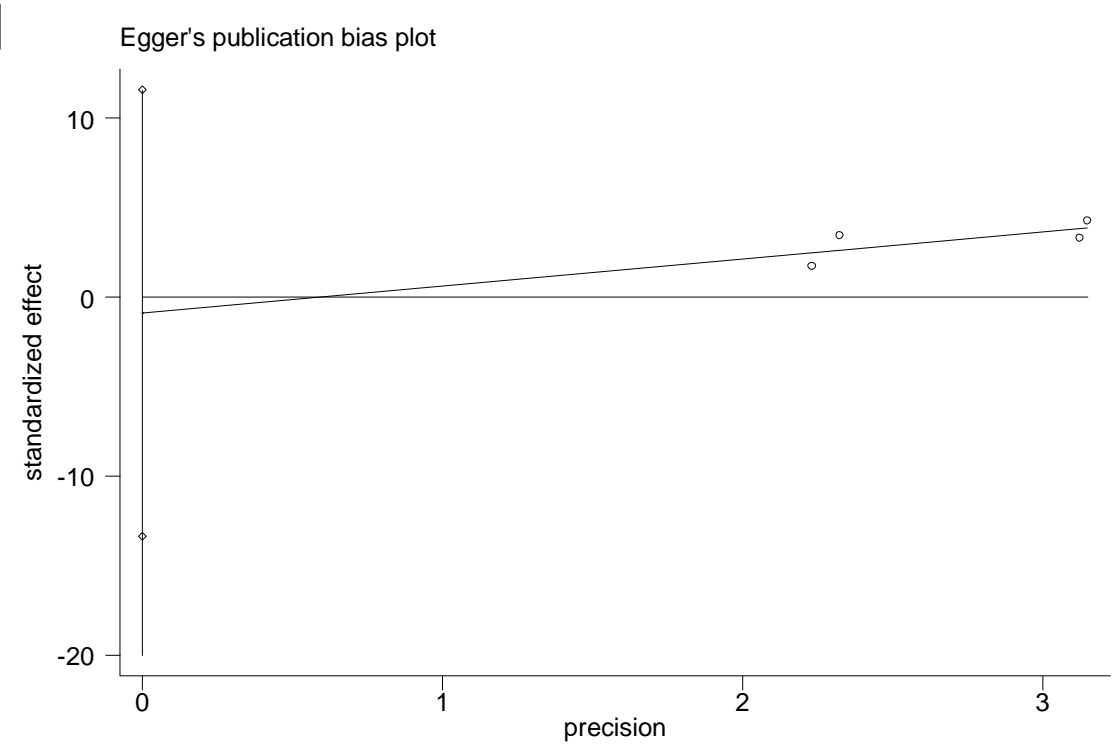

Supplement: Supplementary file 13 [file medi-101-e30942-s013.pdf]

Figure S15 Influence analysis results of DD vs.II

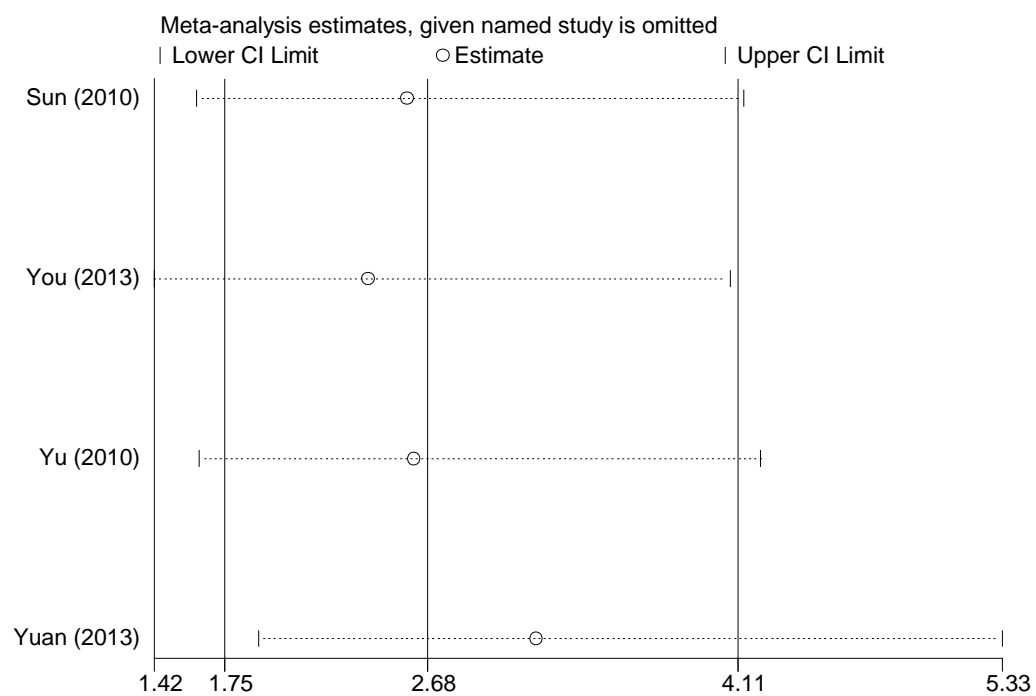

Supplement: Supplementary file 14 [file medi-101-e30942-s014.pdf]

Figure S16 Inverted funnel chart of DD vs.II

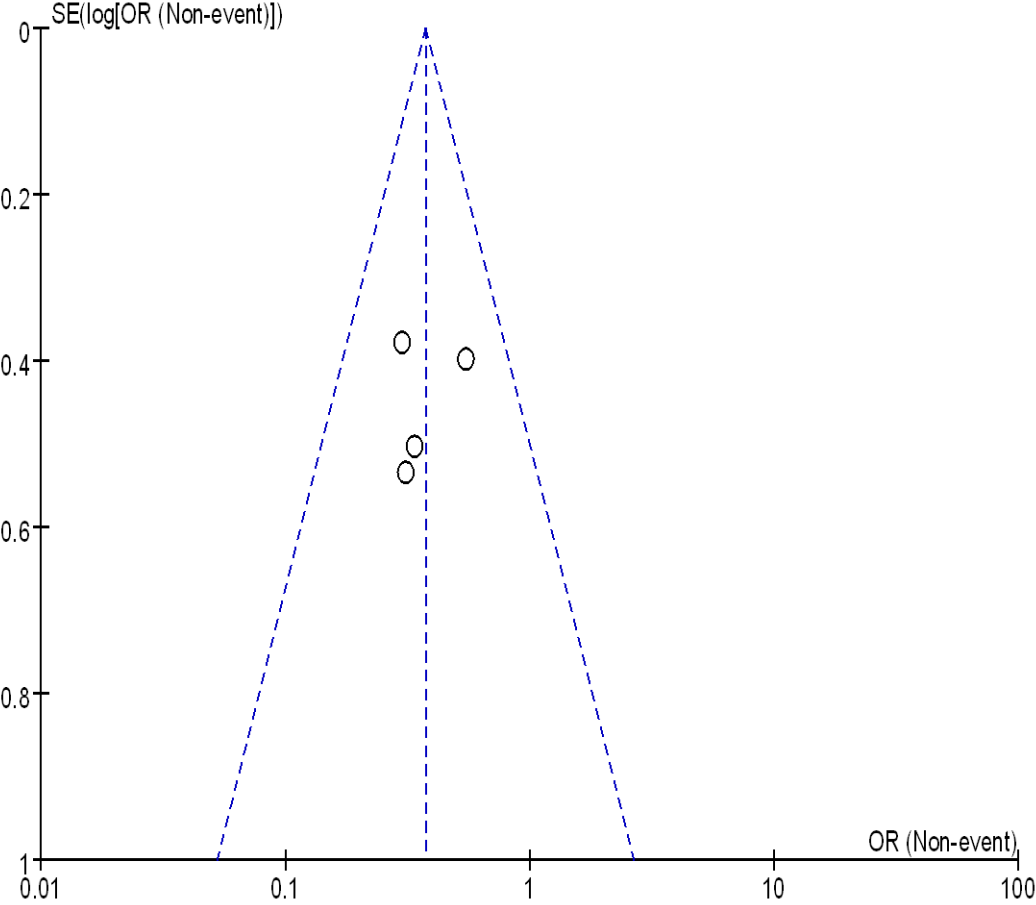

Supplement: Supplementary file 15 [file medi-101-e30942-s015.pdf]

Figure S17 DD vs.II funnel chart generated by Begg's Test

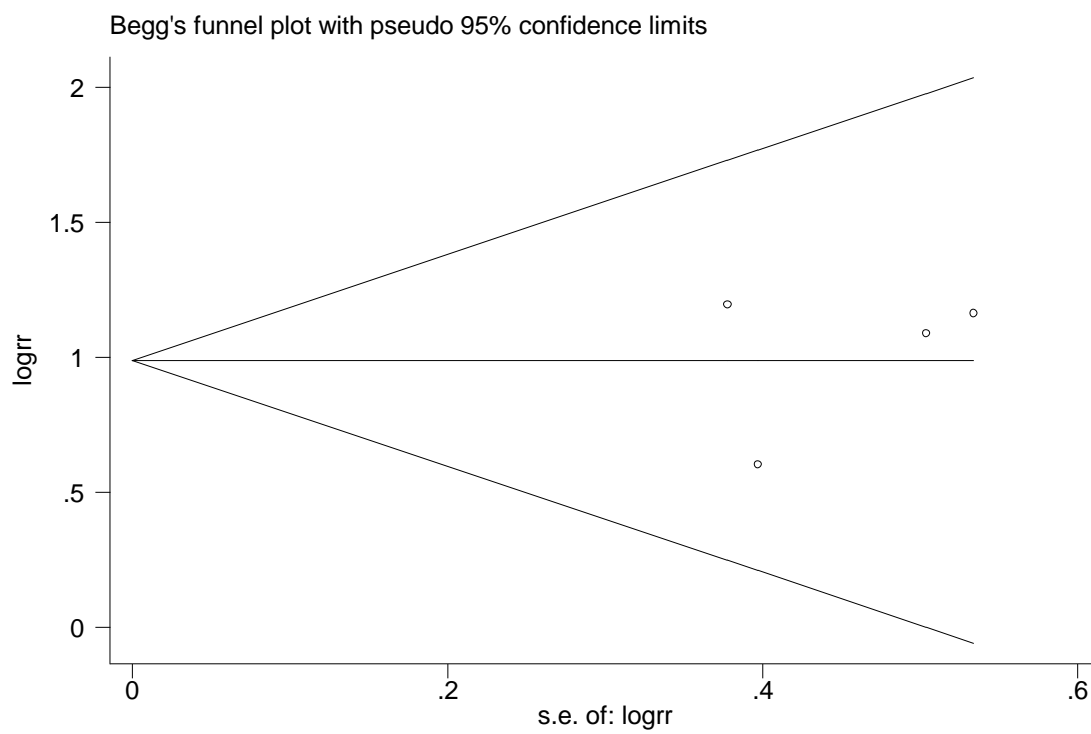

Supplement: Supplementary file 16 [file medi-101-e30942-s016.pdf]

Figure S18 DD vs.II funnel chart of bias generation detected by Egger's test

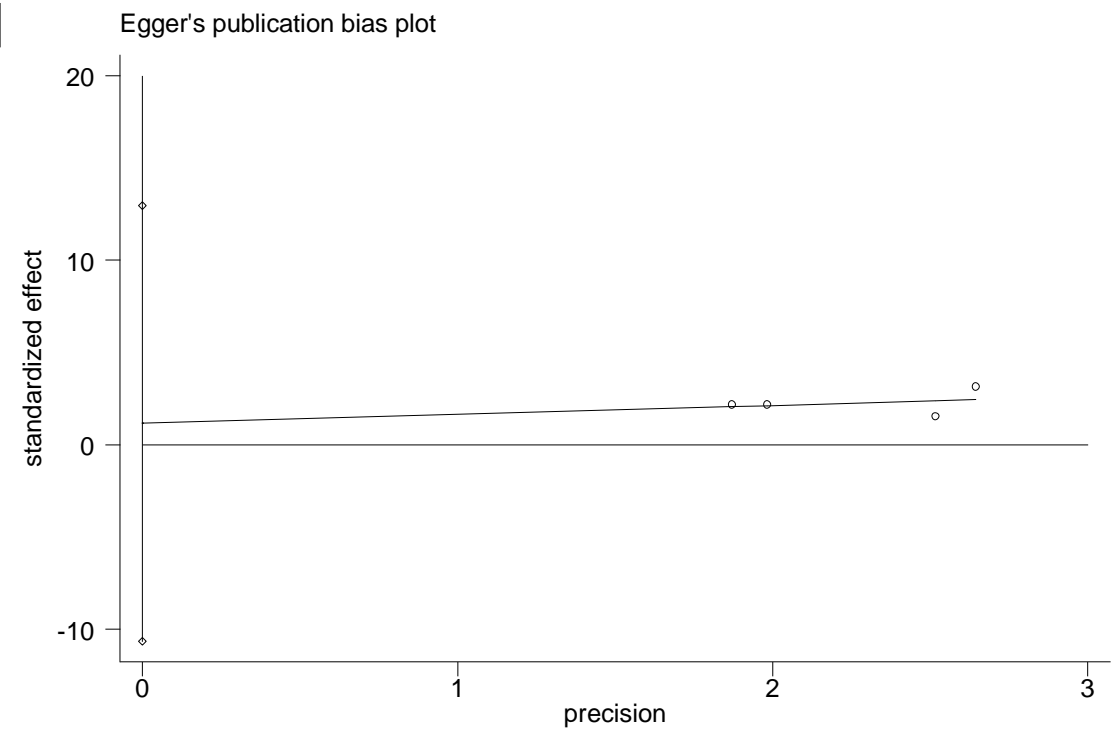

Supplement: Supplementary file 17 [file medi-101-e30942-s017.pdf]

Figure S20 Influence analysis results of ID vs.II

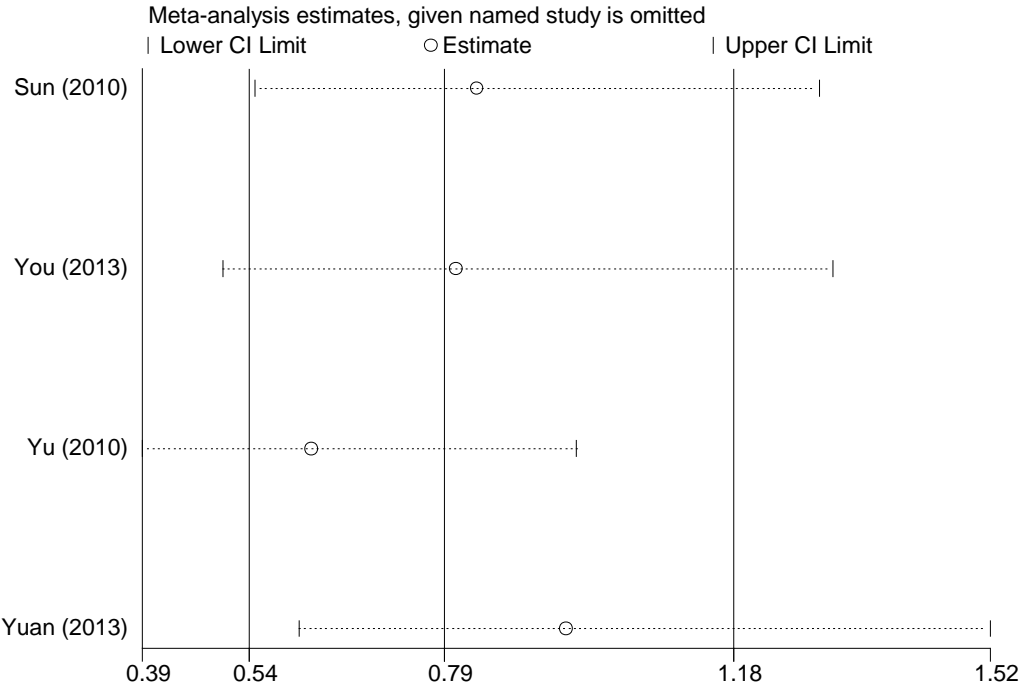

Supplement: Supplementary file 19 [file medi-101-e30942-s019.pdf]

Figure S21 Inverted funnel chart of ID vs.II

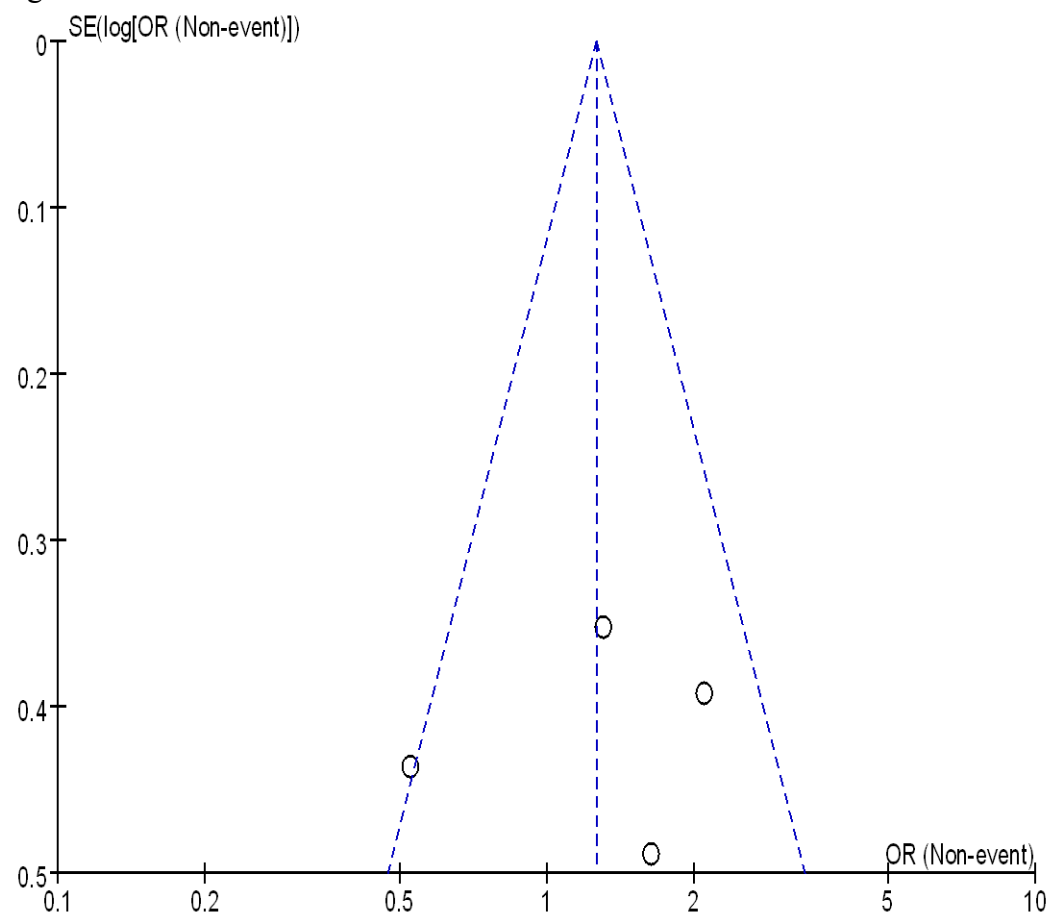

Supplement: Supplementary file 20 [file medi-101-e30942-s020.pdf]

Figure S22 ID vs.II funnel chart generated by Begg's Test

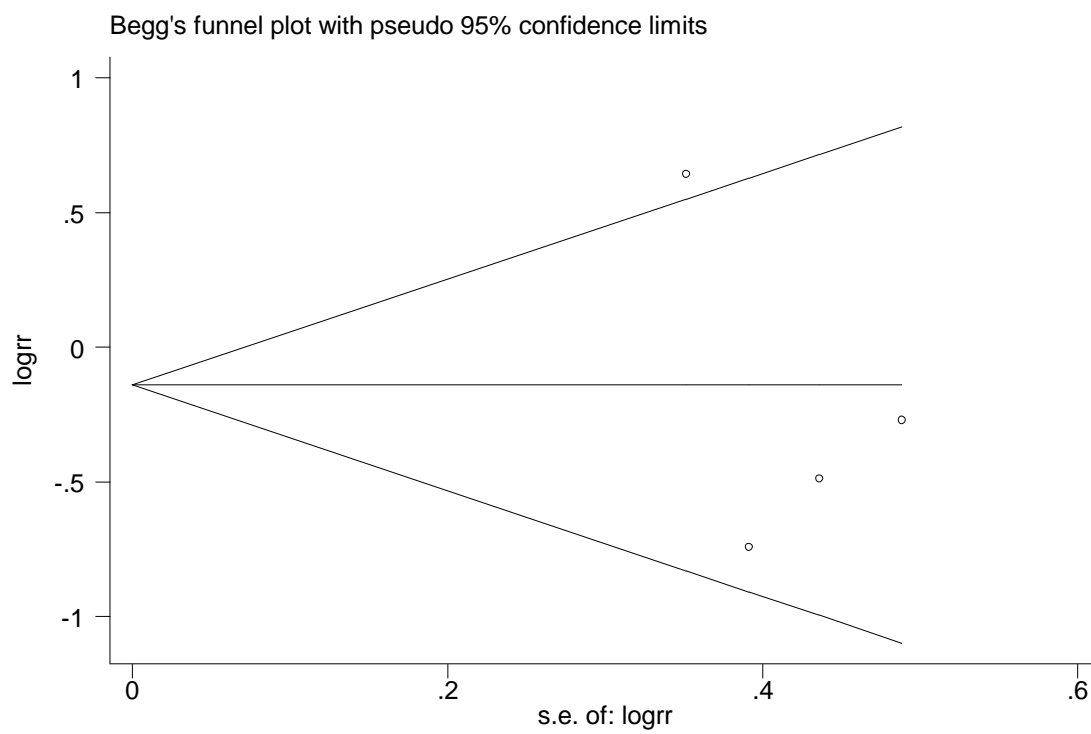

Supplement: Supplementary file 21 [file medi-101-e30942-s021.pdf]

Figure S23 ID vs.II funnel chart of bias generation detected by Egger's test

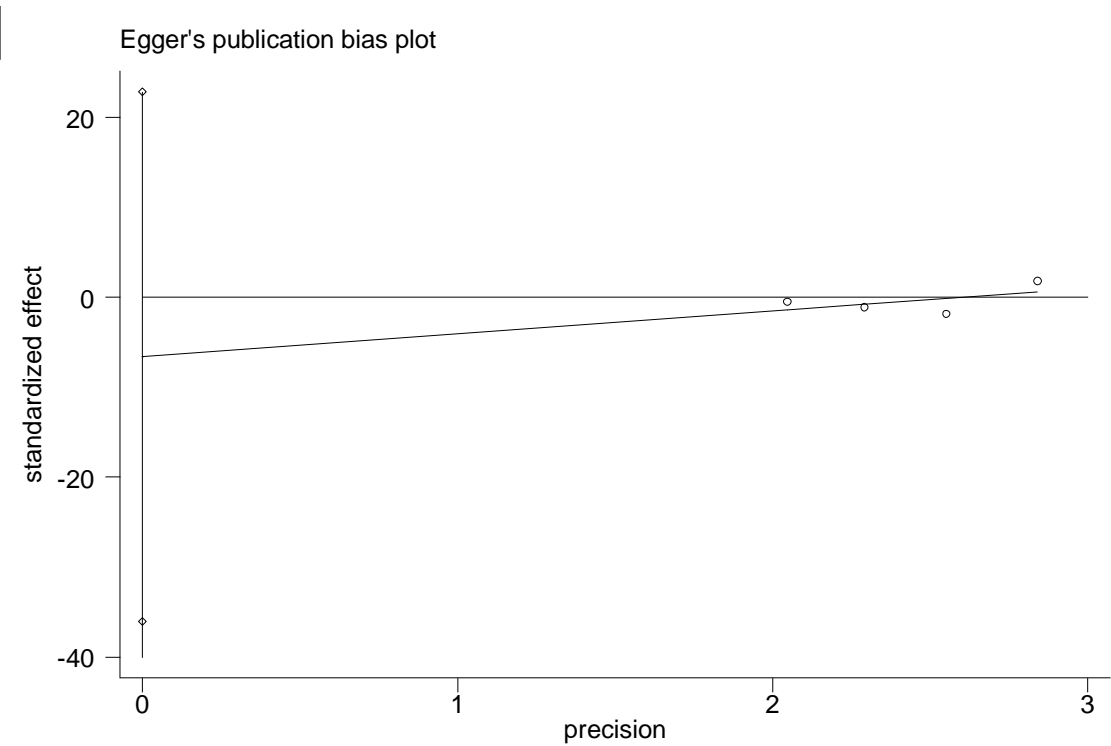

Supplement: Supplementary file 22 [file medi-101-e30942-s022.pdf]
