# Supplementary material for: Increased frequency of angiotensin converting enzyme D allele in Chinese Han patients with idiopathic pulmonary fibrosis: A systematic review and meta-analysis
Source: Medicine (Baltimore). 2022 Oct 7;101(40):e30942. doi: 10.1097/MD.0000000000030942 (PMC9542842; doi:10.1097/MD.0000000000030942)
Supplement: Supplementary file 23 [file medi-101-e30942-s023.pdf]

Table S1 Influence analysis results data of D vs.I

Study omitted	Estimate	[95% Conf.	Interval]
Sun (2010)	1.8375818	1.4301717	2.3610499
You (2013)	1.7901582	1.3565384	2.362385
Yu (2010)	1.8607304	1.4491216	2.3892527
Yuan (2013)	2.0341785	1.5537158	2.6632168
Combined	1.8773682	1.4975001	2.3535967
