# Supplementary material for: Increased frequency of angiotensin converting enzyme D allele in Chinese Han patients with idiopathic pulmonary fibrosis: A systematic review and meta-analysis
Source: Medicine (Baltimore). 2022 Oct 7;101(40):e30942. doi: 10.1097/MD.0000000000030942 (PMC9542842; doi:10.1097/MD.0000000000030942)
Supplement: Supplementary file 24 [file medi-101-e30942-s024.pdf]

Table S2 Detection results of bias in D vs.I by Begg's Test

Begg's Test	
adj. Kendall's Score (P-Q)	2
Std. Dev. of Score	2.94
Number of Studies	4
z	0.68
Pr > |z|	0.497
z	0.34 (continuity corrected)
Pr > |z|	0.734 (continuity corrected)
