# Supplementary material for: Increased frequency of angiotensin converting enzyme D allele in Chinese Han patients with idiopathic pulmonary fibrosis: A systematic review and meta-analysis
Source: Medicine (Baltimore). 2022 Oct 7;101(40):e30942. doi: 10.1097/MD.0000000000030942 (PMC9542842; doi:10.1097/MD.0000000000030942)
Supplement: Supplementary file 25 [file medi-101-e30942-s025.pdf]

Table S3 Detection results of bias in D vs.I by Egger's test

Egger's test						
Std_Eff	Coef.	Std. Err.	t	P>|t|	[95% Conf. Interval]	
slope	0.0589156	0.7155468	0.08	0.942	-3.019834	3.137665
bias	2.665346	3.099901	0.86	0.480	-10.67245	16.00314
