# Supplementary material for: Increased frequency of angiotensin converting enzyme D allele in Chinese Han patients with idiopathic pulmonary fibrosis: A systematic review and meta-analysis
Source: Medicine (Baltimore). 2022 Oct 7;101(40):e30942. doi: 10.1097/MD.0000000000030942 (PMC9542842; doi:10.1097/MD.0000000000030942)
Supplement: Supplementary file 26 [file medi-101-e30942-s026.pdf]

Table S4 Influence analysis results data of DD+ID vs.II

Study omitted	Estimate	[95% Conf.	Interval]
Sun (2010)	1.3849291	0.93848222	2.0437555
You (2013)	1.318163	0.85289288	2.0372474
Yu (2010)	1.1837778	0.79132164	1.7708726
Yuan (2013)	1.5946463	1.0487221	2.4247572
Combined	1.3606475	0.9538487	1.9409386
