# Supplementary material for: Increased frequency of angiotensin converting enzyme D allele in Chinese Han patients with idiopathic pulmonary fibrosis: A systematic review and meta-analysis
Source: Medicine (Baltimore). 2022 Oct 7;101(40):e30942. doi: 10.1097/MD.0000000000030942 (PMC9542842; doi:10.1097/MD.0000000000030942)
Supplement: Supplementary file 28 [file medi-101-e30942-s028.pdf]

Table S6 Detection results of bias in DD+ID vs.II by Egger's test

Egger's test						
Std_Eff	Coef.	Std. Err.	t	P>|t|	[95% Conf. Interval]	
slope	-0.1781792	1.717458	-0.10	0.927	-7.567804	7.211446
bias	1.331152	4.701864	0.28	0.804	-18.89934	21.56164
