# Supplementary material for: Increased frequency of angiotensin converting enzyme D allele in Chinese Han patients with idiopathic pulmonary fibrosis: A systematic review and meta-analysis
Source: Medicine (Baltimore). 2022 Oct 7;101(40):e30942. doi: 10.1097/MD.0000000000030942 (PMC9542842; doi:10.1097/MD.0000000000030942)
Supplement: Supplementary file 29 [file medi-101-e30942-s029.pdf]

Table S7 Influence analysis results data of DD vs. II+ID

Study omitted	Estimate	[95%	Conf. Interval]
Sun (2010)	3.0813756	2.0809674	4.5627217
You (2013)	3.0399382	1.9709227	4.6887808
Yu (2010)	3.5565555	2.4071052	5.2548952
Yuan (2013)	3.5097768	2.2807317	5.4011321
Combined	3.2942251	2.3095114	4.6987943
