# Supplementary material for: Increased frequency of angiotensin converting enzyme D allele in Chinese Han patients with idiopathic pulmonary fibrosis: A systematic review and meta-analysis
Source: Medicine (Baltimore). 2022 Oct 7;101(40):e30942. doi: 10.1097/MD.0000000000030942 (PMC9542842; doi:10.1097/MD.0000000000030942)
Supplement: Supplementary file 30 [file medi-101-e30942-s030.pdf]

Table S8 Detection results of bias in DD vs. II+ID by Begg's Test

Begg's Test	
adj. Kendall's Score (P-Q)	-2
Std. Dev. of Score	2.94
Number of Studies	4
z	-0.68
Pr > |z|	0.497
z	0.34 (continuity corrected)
Pr > |z|	0.734 (continuity corrected)
