# Supplementary material for: Increased frequency of angiotensin converting enzyme D allele in Chinese Han patients with idiopathic pulmonary fibrosis: A systematic review and meta-analysis
Source: Medicine (Baltimore). 2022 Oct 7;101(40):e30942. doi: 10.1097/MD.0000000000030942 (PMC9542842; doi:10.1097/MD.0000000000030942)
Supplement: Supplementary file 31 [file medi-101-e30942-s031.pdf]

Table S9 Detection results of bias in DD vs. II+ID by Egger's test

Egger's test						
Std_Eff	Coef.	Std. Err.	t	P>|t|	[95% Conf. Interval]	
slope	1.506818	1.058437	1.42	0.291	-3.047269	6.060905
bias	-0.8946964	2.900335	-0.31	0.787	-13.37383	11.58444
