# Supplementary material for: Increased frequency of angiotensin converting enzyme D allele in Chinese Han patients with idiopathic pulmonary fibrosis: A systematic review and meta-analysis
Source: Medicine (Baltimore). 2022 Oct 7;101(40):e30942. doi: 10.1097/MD.0000000000030942 (PMC9542842; doi:10.1097/MD.0000000000030942)
Supplement: Supplementary file 32 [file medi-101-e30942-s032.pdf]

Table S10 Influence analysis results data of DD vs.II

Study omitted	Estimate	[95% Conf.	Interval]
Sun (2010)	2.5876725	1.6176962	4.1392498
You (2013)	2.4087479	1.4223487	4.079215
Yu (2010)	2.6211398	1.629565	4.2160788
Yuan (2013)	3.1847494	1.9020551	5.3324575
Combined	2.6806892	1.746484	4.1146067
