# Supplementary material for: Increased frequency of angiotensin converting enzyme D allele in Chinese Han patients with idiopathic pulmonary fibrosis: A systematic review and meta-analysis
Source: Medicine (Baltimore). 2022 Oct 7;101(40):e30942. doi: 10.1097/MD.0000000000030942 (PMC9542842; doi:10.1097/MD.0000000000030942)
Supplement: Supplementary file 34 [file medi-101-e30942-s034.pdf]

Table S12 Detection results of bias in DD vs.II by Egger's test

Egger's test						
Std_Eff	Coef.	Std. Err.	t	P>|t|	[95% Conf. Interval]	
slope	0.4871237	1.202413	0.41	0.725	-4.686443	5.66069
bias	1.154195	2.741085	0.42	0.715	-10.63974	12.94813
