# Supplementary material for: Increased frequency of angiotensin converting enzyme D allele in Chinese Han patients with idiopathic pulmonary fibrosis: A systematic review and meta-analysis
Source: Medicine (Baltimore). 2022 Oct 7;101(40):e30942. doi: 10.1097/MD.0000000000030942 (PMC9542842; doi:10.1097/MD.0000000000030942)
Supplement: Supplementary file 35 [file medi-101-e30942-s035.pdf]

Table S13 Influence analysis results data of ID vs.II

Study omitted	Estimate	[95%	Conf. Interval]
Sun (2010)	0.83735746	0.54271019	1.2919742
You (2013)	0.80934191	0.50008571	1.309844
Yu (2010)	0.61759162	0.39332989	0.96971887
Yuan (2013)	0.95585102	0.60142267	1.5191499
Combined	0.794189	0.5351456	1.1786253
