# Supplementary material for: Increased frequency of angiotensin converting enzyme D allele in Chinese Han patients with idiopathic pulmonary fibrosis: A systematic review and meta-analysis
Source: Medicine (Baltimore). 2022 Oct 7;101(40):e30942. doi: 10.1097/MD.0000000000030942 (PMC9542842; doi:10.1097/MD.0000000000030942)
Supplement: Supplementary file 37 [file medi-101-e30942-s037.pdf]

Table S15 Detection results of bias in ID vs.II by Egger's test

Egger's test						
Std_Eff	Coef.	Std. Err.	t	P>|t|	[95% Conf. Interval]	
slope	2.534985	2.793178	0.91	0.460	-9.48309	14.55306
bias	-6.606742	6.84739	-0.96	0.436	-36.06868	22.8552
